# Assessment and optimisation of regional scale wind farm deployment using machine learning

**DOI:** 10.1038/s44172-026-00673-w

**Published:** 2026-04-29

**Authors:** Simon C. Warder, Mariana C. A. Clare, B. Bhaskaran, Matthew D. Piggott

**Affiliations:** 1 Department of Earth Science and Engineering, Imperial College London, London, UK; 2 Shell Information Technology International Ltd, Shell Centre, London, UK

**Keywords:** Wind energy, Computational science

## Abstract

The impact of inter-farm wakes is a growing issue as offshore wind is scaled up to meet renewable energy needs. High-fidelity simulations which capture such wake effects under potential future build-out scenarios are required to enable regional-scale planning which can mitigate wake impacts. Here, we present a machine learning-based workflow for estimating power losses due to inter-farm wake effects, suited to efficient analysis and optimal planning of future build-out. We apply this tool to the assessment of planned build-out in the North Sea. We estimate that percentage power losses due to inter-farm wakes will more than double compared with their current level, reaching 2.4%, and that increased losses in summer will exacerbate natural seasonal variability in resource. Our tool also facilitates sensitivity analysis and optimisation of wind farm fleets with respect to a variety of design choices. In this work we optimise total fleet power output with respect to small adjustments in future farm locations, finding that wake-induced losses can be reduced by one third via careful spatial planning, corresponding to annual economic gains of £160m compared with current plans.

## Abstract (web summary)

Wind farm wakes are a growing issue as offshore wind is scaled up, and their mitigation requires careful regional-scale planning. Simon C. Warder and colleagues present a machine learning modelling tool enabling efficient optimisation of wind farm build-out.

## Introduction

The build-out of offshore wind farms (OWFs) in the North Sea is among the most rapid worldwide, and is expected to accelerate over the next decades to meet climate goals. By the end of 2022, Europe’s total OWF installed capacity (IC) exceeded 30 GW^1^, with 22 GW installed in the North Sea alone^2^. This will need to increase twofold by 2030 and tenfold by 2050 in order to meet EU targets of 60 and 300 GW respectively^3^. Similar UK targets will require increasing 14 GW of currently installed OWF capacity^1^ to 50 GW by 2030^4^. This will require a rapid increase in the size and density of OWFs in European waters such as the North Sea.

Wakes arise due to the removal of kinetic energy from the atmosphere by wind turbines, and are known to impact OWF energy yields. In this work we focus on inter-farm wakes, which arise due to the cumulative wake effect across entire farms, impacting other farms nearby. Such farm-induced wakes have been observed to persist 10s of km downstream of OWFs^5–8^, depending on many factors including wind speed and atmospheric stratification^9,10^. This results in the reduction of OWF energy yields and increased costs^11–13^. Inter-farm interactions have implications for industry and governmental needs, including the optimal design of individual farms, short- to medium-term power predictions for load balancing purposes or day-ahead energy markets, and at a larger (e.g. regional/multi-decadal) scale for planning future OWF build-out. We focus on this latter application within the present study, although the tools we introduce have broad applications.

Future economic losses due to large-scale OWF deployment are dependent on the build-out trajectory; several studies have highlighted this issue, suggesting that future build-out should be coordinated across developers and countries^6,13–15^. There is also insufficient legal structure protecting OWF developers from adverse wake effects impacting their farms^16,17^. The need to consider inter-farm interactions will likely increase in future, given the scale of planned installations and the trends in the size and density of turbines and farms^18–20^.

Despite consensus on the need for coordinated build-out^6,15,17^, to date there has been a lack of tools well suited to wind farm spatial planning accounting for inter-farm wake effects. Mesoscale numerical weather prediction (NWP) models are considered to be the only realistic option for the analysis of multiple wind farm wakes at regional scales^21^. A particularly popular choice is the Weather Research and Forecasting (WRF) model^22^, which includes a choice of wind farm parameterisations^23–28^, and has been extensively studied in the literature in the context of wind farm wake modelling^13,29–33^. However, the computational cost of NWP models is a barrier to the assessment of build-out scenarios on regional and multi-decadal scales. Most numerical studies of inter-farm wake effects are limited to small spatial scales^13,29^ and/or the selection of a small number of weather conditions^31,32,34^. Akhtar et al.^19^ do undertake a thorough regional-scale numerical investigation over a decadal time period, exploring energy production limits in the North Sea based on planned OWF build-out. However, this study is limited to the assessment of a single build-out scenario. Dörenkämper et al.^35^ investigated wake losses across the North Sea under future build-out scenarios, although this study is limited to a single representative year of weather conditions, and results are presented from only five fixed build-out stages. Thus, there is an unmet need for computationally efficient tools well suited to regional-scale multi-scenario assessment, quantification of uncertainty due to unknown future build-out, sensitivity analysis, and optimisation.

The related problem of intra-farm wake effects is better studied, and efficient approaches have been developed for predicting wakes from individual turbines. So-called engineering wake models have been developed for this purpose, such as those available within the tools PyWake^36^ and FLOw Redirection and Induction in Steady State (FLORIS)^37^. These typically rely on simple analytic models or lookup tables using datasets generated from high-fidelity numerical simulations. Machine learning approaches to turbine wake modelling are also growing in popularity, typically trained using high-fidelity numerical models^38–42^. Although well suited to layout optimisation for individual farms, such turbine wake models cannot be straightforwardly applied to the regional-scale problem considered within this study. Engineering models typically suffer from excessive wake decay at farm-to-farm length scales^13,43^, underestimating inter-farm wake-induced losses. Furthermore, since both analytic and data-driven approaches have been designed for intra-farm scale problems, such models typically neglect important mesoscale flow variations^44^, often assuming uniform flow and accounting for very few input parameters (often only wind speed and turbulence intensity). Thus even engineering wake models designed to capture wake decay over longer distances, such as the recently-developed TurbOPark model^45,46^, still require assumptions which are typically invalid for real-world conditions at farm-to-farm scales. Although computationally efficient, these existing tools cannot therefore be straightforwardly applied to regional-scale build-out analysis and spatial planning.

The present study introduces a machine learning-based tool for predicting inter-farm wakes in real-world weather conditions. The tool requires a ‘background’ (i.e. farm-free) NWP simulation for each time period of interest, subsequently utilising a neural network to predict the wakes from OWFs placed within this background wind field. The power production of farms within a wake-affected wind field can then be straightforwardly predicted. Requiring only one farm-free NWP run, this framework drastically reduces the computational cost associated with assessing multiple build-out scenarios/stages, or varying farm locations or designs, while accounting for inter-farm wake effects. This enables analysis and optimisation of OWF fleet deployments over regional and multi-decadal scales.

Within this work, we employ the machine learning tool to analyse planned build-out in the North Sea. This analysis is based on weather conditions spanning 20 years (2000–2019), and incorporating real planned OWF build-out over the next several years. We further investigate specific losses due to interactions between individual pairs of farms, to gain insight into the characteristics of OWF fleets which give rise to these losses. Finally, we demonstrate the application of the workflow to spatial planning, in order to mitigate wake-induced losses. To do this, we perform sensitivity analysis of future OWF fleet performance with respect to perturbations in the locations of farms, and apply a genetic algorithm to find the optimal configuration. Such an optimisation of OWF fleets over regional/multi-decadal scales is made possible only by the rapid nature of our machine learning approach.

## Results

This work considers currently operational OWFs in the North Sea, as well as planned build-out over the next several years^2^. The focus of this work is on inter-farm effects. Before we can proceed with a study of inter-farm effects, we must first define a distinction between inter- and intra-farm scales. With a very small number of exceptions (which we discuss in Supplementary Information section S-2), the criterion we use is that any two farms with a separation distance of greater than 2 km or 12 rotor diameters are treated as separate entities (and their interactions regarded as inter-farm), and any pairs of farms not satisfying either condition are grouped together (and their interactions regarded as intra-farm). Broadly, this ensures that farm groups which form closely tessellated clusters are treated as single entities, while uncoordinated developments are treated as separate. We acknowledge that this criterion is somewhat arbitrary, but it is a necessary distinction to make, since the total losses due to inter-farm wake effects will be sensitive to this definition. The use of this criterion means that our results are not dependent on how each country or developer has subdivided their lease areas.

**Fig. 1:**
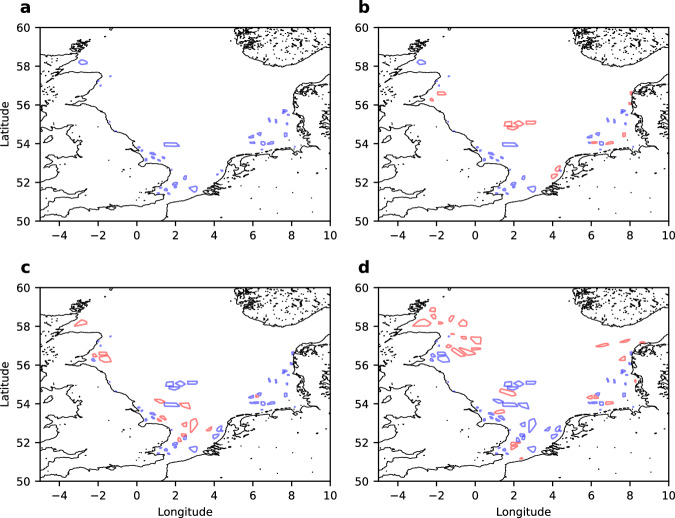
Maps of the build-out stages studied within this work. Build-out stages A–D, as described in Table 1, are depicted in **a**–**d**, respectively. Blue polygons indicate farms or lease areas unchanged from the previous build-out stage. Red polygons indicate newly added farms or those extended compared to previous stages. Geographic data within this figure is derived from the MODIS land use dataset^58,59^, which is an open access dataset and distributed as static geographic data via the WRF Preprocessing System (WPS)^57^.

As shown in Fig. 1 and Table 1, the farms are divided into four incremental build-out stages. Our performance analysis of each build-out stage is based on instantaneous weather conditions spaced 5.25 days apart, spanning 2000–2019 inclusive (1392 evenly-spaced snapshots). Selecting a 20-year period ensures that a fully representative range of weather conditions is included. The 5.25-day spacing is chosen due to computational cost considerations. 5.25 days is of the same order of magnitude as the typical wind speed autocorrelation length in the region, hence the value in sampling at higher frequency is limited. We choose a non-integer period to avoid any bias towards a particular atmospheric stability regime, which typically follows a daily cycle and is known to impact wake extent^9^.

**Table 1:** Build-out stages used within this work, based on the planning stage of each farm

Stage label	Description	Range of (expected) commission dates
A	Fully commissioned by end of 2022	2002–2022
B	+ partial generation or under construction	2023–2026
C	+ consent authorised or application submitted	2025–2031
D	+ concept/early planning	2027–2035

There is some overlap between expected commission date ranges for each build-out stage. However, since these dates are not available for all farms, we choose to group by planning stage. Each build-out stage analysed within this work includes all farms from previous stages.

### Build-out analysis

**Fig. 2:**
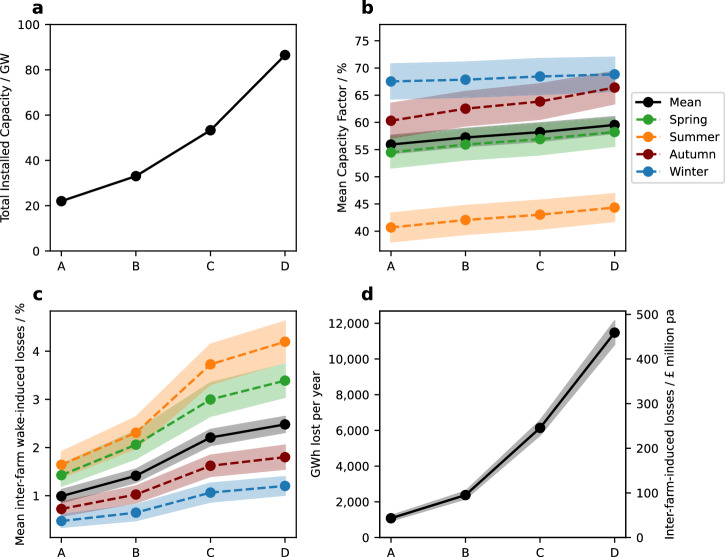
Analysis of planned build-out stages. **a** Total installed capacity (IC), **b** capacity factor (CF), **c** losses due to inter-farm wake interactions as a percentage of total annual energy, **d** total annual losses in both GW h, and estimated economic losses based on an assumed price of electricity of £40 per MW h. Shaded regions indicate 95% confidence intervals, accounting for variation in wind conditions and estimated modelling errors.

For each build-out stage, we employ our neural network workflow to estimate the mean power output from each farm, over the 20-year analysis period. We do this both with and without inter-farm wakes, and thus estimate the wake-induced power losses, which we can compare between build-out stages. Figure 2 shows the evolution in total IC, mean capacity factor (CF), mean percentage losses due to inter-farm wakes, and total annual energy lost to wake interactions, by build-out stage, and with seasonal breakdowns.

Figure 2a shows that total IC is projected to increase fourfold, from 22 GW at stage A, to 87 GW at stage D. The mean CF is projected to increase by 3.4%, as indicated in Fig. 2b. This is primarily because, as shown in Fig. 1, most new deployment in the latter build-out stages is towards the north, where the underlying wind resource is slightly higher (see Supplementary Information section S-1). We note that the CFs presented here are overestimated due to estimating via a simple power curve approach, neglecting intra-farm wake effects. The focus of this work is on inter-farm effects. We found via preliminary experiments, which can be found in section S-9 of the Supplementary Information, that our estimated inter-farm impacts do not change if we additionally account for intra-farm losses via models such as PyWake, while the computational cost of our analysis increases substantially. In light of these results, we choose to use the simple power curve approach.

Figure 2c shows that mean losses due to inter-farm wake effects, as a percentage of annual energy production, are projected to increase substantially from stages A to D, from 0.98% to 2.44%. This is due to decreasing separation distances between OWFs, and demonstrates a saturation effect where increasing capacity leads to diminishing returns. This is reflected in the rapid increase in total annual energy yield which is lost to wake effects, increasing tenfold as shown in Fig. 2d. Assuming a price of £40 per MW h (a reasonable estimate of typical wholesale prices^47^ but lower than estimates of levelised cost of electricity from European offshore wind^48^), annual economic losses due to inter-farm wake effects in the North Sea are estimated as £44m for the current fleet, rising to £461m by build-out stage D.

Looking at the trends for individual seasons, we observe the lowest CFs in the summer, when wind speeds are lower on average. This also leads to greater relative wake-induced losses, with summer losses around five times greater than in winter. Power output is only sensitive to wake effects when the wind speed is below turbine rated speeds. This is more likely in summer, hence the greater relative impact of inter-farm wakes. These effects therefore exacerbate seasonal variations in wind power.

**Fig. 3:**
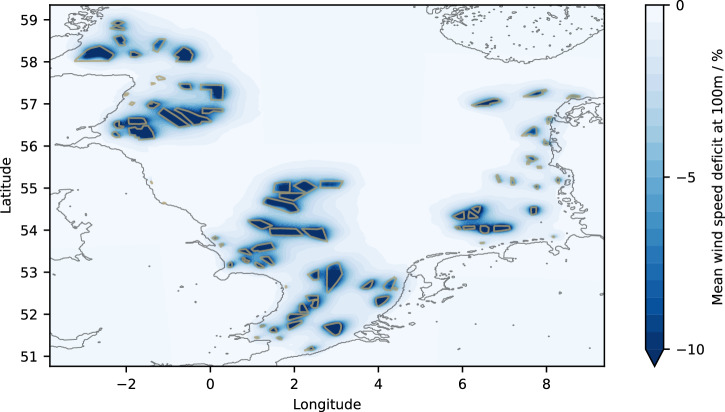
Map of percentage wake-induced wind speed deficits throughout the study region, for build-out stage D. Geographic data within this figure is derived from the MODIS land use dataset^58,59^, which is an open access dataset and distributed as static geographic data via the WRF Preprocessing System (WPS)^57^.

**Fig. 4:**
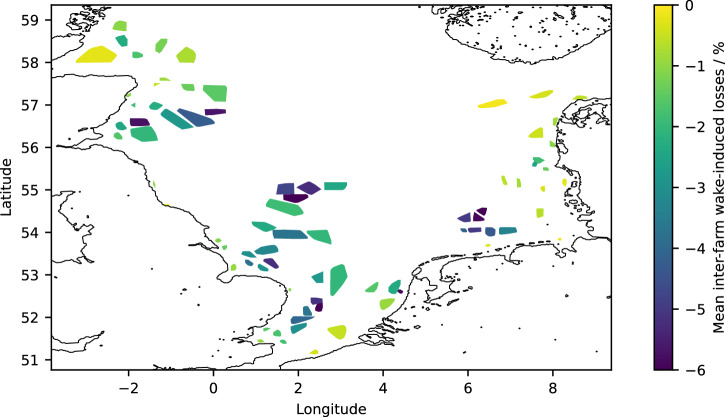
Map of mean inter-farm wake-induced power losses throughout the study region, for build-out stage D. Geographic data within this figure is derived from the MODIS land use dataset^58,59^, which is an open access dataset and distributed as static geographic data via the WRF Preprocessing System (WPS)^57^.

Figure 3 maps the mean percentage wind speed deficit throughout the study region for build-out stage D, modelled using the machine learning framework. Deficits in excess of 10% are typical within wind farms, while mean deficits of around 2–3% are often observed tens of km away from farms. These results are qualitatively consistent with the literature^35^, although the precise build-out scenarios used are different. Figure 4 shows the inter-farm wake-induced power losses, again for build-out stage D. Farms incurring the greatest inter-farm losses are found in regions of dense build-out such as the German Bight (consistent with previous work^20^), as well as the southern North Sea, and offshore East Anglia. In total, 22 out of the 82 farms at this build-out stage incur losses in excess of 3% of AEP, 15 incur losses in excess of 4%, and 7 incur losses in excess of 5%.

### Pair-wise farm interactions

The computational efficiency of our approach allows us to ‘toggle’ individual farms on and off, and observe the resulting power changes at nearby farms. We can thus attribute losses at a given farm to the wake effects from each of its neighbours. To quantify the interaction between a given pair of farms, we define a pairwise percentage loss (PPL) as the inter-farm wake-induced losses induced by one farm on another, as a percentage of its annual energy production. We further define the *mutual* PPL as the total combined losses induced by a pair of farms on each other, as a percentage of their combined annual energy production.

**Fig. 5:**
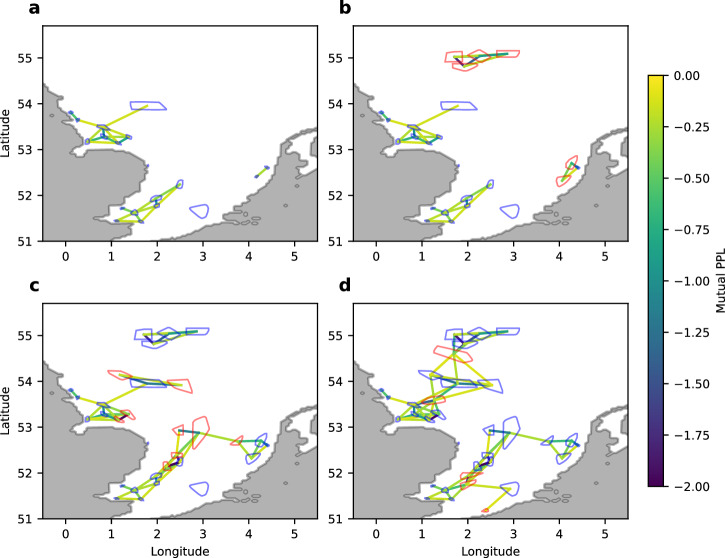
Mutual pairwise percentage losses (PPLs) between individual pairs of farms in the southern North Sea. Build-out stages A–D are depicted in **a**–**d** respectively. As in Fig. 1, red and blue polygons indicate new and unchanged farms, respectively. The colour of the line between each pair of farms indicates the strength of interaction between the two farms. Only mutual PPL values with an absolute value in excess of 0.1% are shown. Geographic data within this figure is derived from the MODIS land use dataset^58,59^, which is an open access dataset and distributed as static geographic data via the WRF Preprocessing System (WPS)^57^.

Figure 5 shows the mutual PPLs for farms in the southern North Sea. Only mutual PPLs with a magnitude of at least 0.1% are shown. At stages A and B, the interactions create small clusters of inter-connected farms. In these scenarios, it may be appropriate to consider the problem of spatial wind farm planning as a series of disconnected smaller-scale problems. However, we see for stages C and D that as more farms are built, the clusters expand and become connected. By stage D, disconnected clusters still arise, but span larger spatial scales. It is clear that with future build-out, it will not be possible to consider small regions independently, and the optimisation of resource extraction becomes an increasingly complex problem involving more farms and larger spatial scales. This further highlights the need for low-cost modelling tools to support design and planning.

**Fig. 6:**
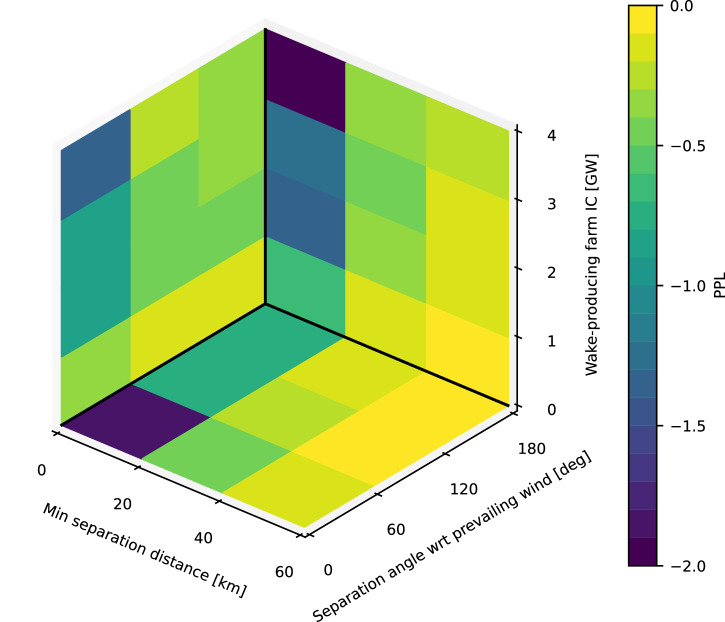
Orthogonal slice plot showing the variation in mean pairwise percentage loss (PPL) with farm separation distance, separation angle with respect to the prevailing wind, and IC of the wake-inducing farm, for build-out stage D. PPL decreases rapidly with inter-farm distance, and is greater for farms situated downstream in the prevailing wind direction. Losses also increase with the IC of the wake-inducing farm.

We can also use the PPL to investigate the factors which produce the greatest wake-induced losses. Figure 6 shows the PPL for all farms at build-out stage D, as a function of minimum inter-farm separation distance, separation angle with respect to the local prevailing wind, and the IC of the wake-producing farm. Losses decrease rapidly as separation distance increases and are approximately zero for distances greater than 50 km. This is consistent with typical wake interaction distances from the existing literature^5–7,13,16^. Through a linear regression, we find that separation distance explains 35% of the variance in PPL values between farms separated by less than 60 km. The PPL also depends on the separation angle between the two farms. For a given pair of farms, one which is positioned downstream with respect to the prevailing wind (i.e. a relative separation angle closer to zero) incurs greater losses than one positioned upstream (separation angle 180^∘^). Although weaker than separation distance, the influence of separation angle is nevertheless statistically significant, explaining 7% of the variance in the PPL. Finally, the greater the IC of the wake-inducing farm, the greater the losses experienced. This explains a further 6% of the variance.

### Spatial planning: sensitivity analysis & optimisation

It is widely acknowledged that future OWF developments must be coordinated in order to mitigate losses from inter-farm wakes. Our neural network-based workflow can support such coordinated planning, owing to the low computational cost associated with its repeated application to a large number of possible scenarios (e.g. farm designs or locations).

Here, we apply the tool to sensitivity analysis and optimisation of OWF fleet performance, with respect to the precise location of future farms. By build-out stage D, there are 82 farms within our case study region. We hold fixed the 40 existing operational farms from build-out stage A, along with any planned future extensions to these farms, but we assume all 42 future planned farms (from build-out stages B–D) can be adjusted from their planned locations, by up to 12 km. To simplify the problem we constrain the farm perturbations to a fixed set, which is selected a priori. We select four possible displacement distances (3.5, 7, 10 and 12 km), and four displacement directions; see Fig. S-10 in the Supplementary Information. Including the unperturbed farm location, there are 17 possible locations for each farm.

To assess the sensitivity of OWF fleet performance to precise farm locations, we sample from the set of possible farm location combinations. There are 42^17^ possible combinations, from which we draw 1000 random samples, re-drawing any samples which result in overlapping farms. For each sample, we apply the neural network workflow to compute three quantities:

- The ‘wake-free’ mean total power output from the fleet (turning off all inter-farm wakes),
- The ‘waked’ mean total power output from the fleet (turning on inter-farm wakes),
- The percentage power losses due to inter-farm wakes (i.e. the % difference between (a) and (b)).

**Fig. 7:**
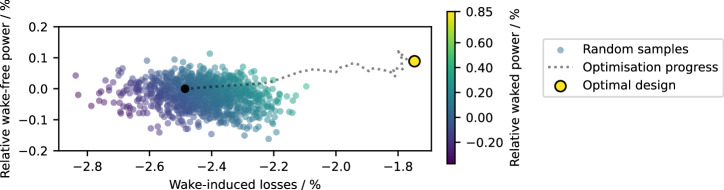
Scatter plot showing the relationship between the wake-free power, the wake-induced losses, and the waked power, for 1000 random samples. Powers are measured as percentage changes relative to the baseline design. The random samples have relative waked power values of between -0.37 and 0.41%. The optimal design (solid circle, on same colour scale) substantially outperforms any of the randomly sampled designs with a relative waked power of 0.85%, driven mainly by a reduction in wake-induced losses.

For (a) and (b) we present values as percentage changes relative to the unperturbed (or baseline) fleet. Figure 7 presents the values of the above three quantities, for the random samples. The background power varies by up to 0.16% from the baseline background power. Wake-induced losses vary between -2.84% and -2.10%. The resulting net power varies by up to 0.41%.

We then apply a genetic optimisation algorithm^49^ to find the configuration which maximises the total power output of the entire OWF fleet. Genetic algorithms have been frequently applied to the similar topic of wind farm turbine layout optimisation^50,51^, and are a natural choice for our problem, owing to its large search space and the existence of local minima/maxima.

The dotted line in Fig. 7 shows the progress of the genetic optimisation algorithm after each generation. The solid circle indicates the optimal solution, which substantially outperforms any build-out design selected within the random sample, achieving an improvement of 0.85% in net power. This is driven by decreased wake-induced losses, and to a slightly lesser extent, by increased wake-free power. Note that this increase in power is around one third of the baseline wake-induced losses (estimated above as 2.44% for build-out stage D). Again assuming a price of £40 per MW h, this corresponds to annual economic gains of £160m after optimisation. This shows that there is substantial potential to mitigate wake-induced losses via intelligent spatial planning.

**Fig. 8:**
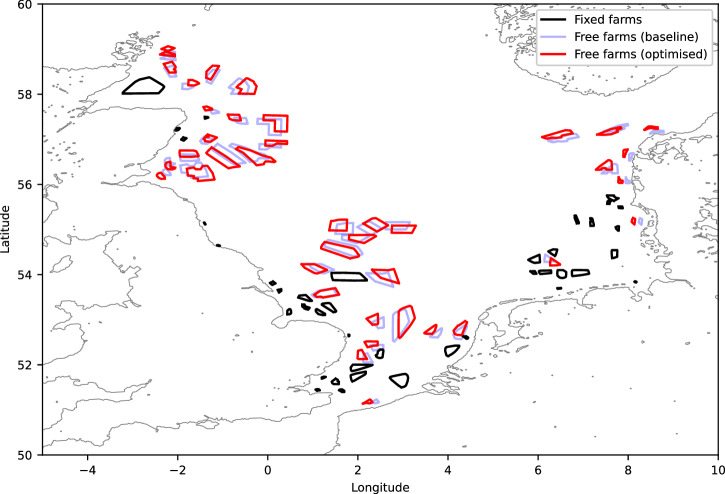
Farm locations before and after optimisation. In general, the optimisation acts to spread farms out spatially, and/or migrate slightly further offshore. Geographic data within this figure is derived from the MODIS land use dataset^58,59^, which is an open access dataset and distributed as static geographic data via the WRF Preprocessing System (WPS)^57^.

Figure 8 shows the locations of all farms in this case study, before and after optimisation. In general, it can be observed that farms are slightly spread out after optimisation. In the east of the model domain, offshore Denmark, there is also a slight trend towards increasing offshore distances, exploiting the coastal gradient in wind speeds. This is consistent with the increase in wake-free power achieved after optimisation, noted above. These results are consistent with intuition, lending confidence to the methodology.

Of the 82 farms in the North Sea fleet, 69 produce greater power in the optimised design than in the baseline design, with 21 of these increasing output by at least 1%. Only 13 farms produce less power after optimisation, and just six farms decrease output by more than 0.1%. However, it is noteworthy that 12 of the 40 existing farms (held fixed in this optimisation) suffer decreased power output after optimisation, compared with only one of the 42 new farms. Optimising overall yield of the future OWF fleet therefore requires accepting adverse effects for a substantial fraction of the currently operational farms.

The results from an additional case study focused on the southern North Sea can be found in Section S-8 in the Supplementary Information, including validation of the results using full NWP modelling.

## Discussion

We have presented results from a machine learning-based workflow for estimating OWF power losses due to inter-farm wake effects. This workflow constitutes a new tool for build-out analysis and spatial planning, which are of growing significance given the current rate of wind farm development. The tool drastically reduces the computational cost associated with such analysis, since it relies only on a single set of ‘background’ (farm-free) NWP model simulations, and can subsequently be used to estimate losses associated with a large number of build-out scenarios at minimal further computational cost.

Using this tool, we predict that the currently deployed OWF fleet in the North Sea incurs mean inter-farm wake-induced losses of 0.98%, but that this will increase to 2.44% given planned build-out over the next decade, with 15 out of 82 farms experiencing losses in excess of 4%. These losses should be interpreted in the context of increasingly narrow profit margins within the industry^52^, the uncertainty posed to developers given that future build-out is unknown, and the attention inter-farm wakes currently receive within planning and consenting processes^53^. It should also be emphasised that these are mean losses, with instantaneous losses often far greater in magnitude. Assuming a price of £40 per MW h, our methodology estimates annual economic losses of £44m for current farms, rising to £461m per annum for planned build-out. The results indicate that wake-induced losses are around five times greater in summer than in winter, exacerbating the seasonal variation in wind power production. In future work we will consider possible longer-term build-out trajectories, which may further require the consideration of climate uncertainties.

This study has also presented an example application of the tool to sensitivity analysis and optimisation. By randomly perturbing the precise locations of planned future farms by up to 12 km, total mean power varies by up to 0.41%. By optimising the precise locations of future OWF developments via a genetic algorithm, we can increase total mean power by 0.85% compared with the current planned build-out. This corresponds to estimated economic savings of approximately £160m per annum, i.e. reducing the predicted losses by around one third. This demonstrates the substantial potential for the mitigation of wake-induced losses via careful spatial planning. The optimisation resulted in 21 of the 82 farms increasing their mean power output by more than 1%, although 12 of the 40 existing farms produce less power under this optimised scenario. This emphasises the need for co-ordination and co-operation in the design of future build-out.

This analysis has ignored constraints on the locations of OWFs such as bathymetry, shipping lanes or regulatory issues, and has focused on total energy output, ignoring cost considerations which may depend on location. For example, many farms are positioned slightly further offshore after optimisation (see Fig. 8), exploiting the coastal gradient in wind speeds. However, such positioning would likely increase installation, operation and maintenance costs compared with planned locations. However, we have explored only very small perturbations to OWF locations, and neglected the opportunity to adjust their shapes, sizes and array layouts, which would likely enable further opportunities to mitigate wake effects.

Future work will also consider potential developments to other aspects of the modelling workflow. Alternative wake superposition approaches should be explored, as well as improvements to the neural network model itself, such as the inclusion of further geographic or physical fields within the model inputs, including further proxies for atmospheric stability effects. Such inputs may be needed to ensure global generalisability of the approach, with wind climates and resulting wake effects having varied characteristics across the world^54^. Although this study has tested generalisability to unseen regions (the Irish Sea; see Section S-6 of the Supplementary Information), further testing will be required for other regions globally. Generalisation of the approach with respect to spatial resolution would broaden the applicability of the method; this could be achieved via resolution-invariant model architectures such as Fourier Neural Operators. These aspects are beyond the scope of the present study but will be considered in future work.

The presented workflow is suited to analysis and mitigation of wakes at the inter-farm scale, with intra-farm wakes neglected. This was motivated by the use case of regional-scale fleet planning and optimisation, and is consistent with the choice to use the mesoscale WRF model to generate training data, which does not adequately capture wake effects at the intra-farm scale. As demonstrated in Section S-9 of the Supplementary Information, the presented approach can be coupled with intra-farm modelling tools such as PyWake to additionally estimate intra-farm wake losses. The potential for joint farm- and fleet-scale optimisation within this coupled framework requires further research. Future work will also investigate whether the presented machine learning approach can be extended to the intra-farm scale using additional training data from an appropriate high-resolution intra-farm model, enabling direct joint farm- and fleet-scale optimisation.

Nevertheless, the results of this study highlight the value of computationally efficient tools which can be used for the mitigation of inter-farm wake effects via careful spatial planning. The tool introduced within this study has potential applications not only to government agencies for regional-scale spatial planning, but also to developers for robust resource assessment and wind farm design, taking into account the substantial uncertainty introduced by unknown future build-out.

## Methods

### Numerical modelling

Numerical modelling within this study is performed using the Weather Research and Forecasting (WRF) model (v4.4.1)^22^, with a model configuration appropriate to capturing inter-farm wake effects, based on a previously published configuration^13^. The configuration consists of a Europe-wide domain with 18 km grid resolution, with a nested domain over the North Sea at 2 km resolution. Two such nested configurations are used: one for the southern North Sea, and another for the north-west. The two subdomains cover all current and planned wind farms in the North Sea assessed within this study. We use 62 vertical levels, with approximately 10 m resolution near the ground/sea level and covering the rotor area of all turbines we consider. These horizontal and vertical resolutions are consistent with recommendations from the literature^15^. We use a timestep of 30 s for the outer domain, and 10 s for the nested domains. For further detail regarding the model setup, we provide example WRF configuration files in the associated Code Ocean repository^55^. Initial and boundary conditions for the WRF simulations are taken from ERA-5 reanalysis data^56^. Static geographic data were obtained from the WRF Preprocessing System (WPS)^57^, including data from the MODIS land use dataset^58,59^.

Wind farms are based on currently operational installations as well as known lease areas at various stages of development or planning^2^, which we divide into build-out stages A to D, as described in the main text. For each wind farm, WRF requires the locations of all wind turbines and their corresponding power and thrust coefficient curves. Since wind turbine data are not always known (e.g. for future farms, but also due to the proprietary nature of this data), we utilise a set of five ‘standard’ wind turbines with publicly available data^60^, whose rated powers are 2, 5, 8, 10.6, and 15 MW, and assume that wind turbines are placed on a square grid filling the lease area. For each farm, we choose the standard turbine whose rated power most closely matches that of the wind farm data. The turbine spacing is then adjusted so that the overall installed capacity of the modelled farm matches the farm data. These wind farms are then represented within WRF using the Fitch wind turbine parameterisation scheme^24,61^. Each WRF run within this work spans a simulated period of three hours, which sensitivity testing found to be sufficient to spin up a farm wake.

Our choice to employ the WRF model with the Fitch wind farm parameterisation is based on its prevalence in the literature, where it has been well studied in the context of wind farm wake modelling^13,29–33^, and has been shown to perform well when benchmarked against the alternative parameterisations available within WRF^23^. One limitation of such parameterisations is that the influence of the precise turbine layout on the formation of the farm-scale wake is neglected, since the positions of turbines within WRF grid cells are not resolved. However, WRF with the Fitch scheme has been shown to give good agreement with observations^13,32,33^ and is thus considered adequate for use within this study. We note also that the workflow we present can be readily applied to any alternative NWP model or parameterisation scheme. However, this is left to future work.

### Machine learning workflow

The crux of the machine learning workflow is the development of a neural network model for predicting farm-induced wake deficit fields, based on ‘background’ wind fields from a WRF simulation, in addition to data regarding the turbine type and layout comprising the farm. For a given farm, the wakes from its neighbours are predicted using this neural network, superposed, and combined with the background wind field to produce a wake-affected wind field. This in turn is used as input to a farm-scale model for estimating the power output of a farm; in this work we apply turbine power curves to the predicted wind field, but this could be replaced with an off-the-shelf intra-farm wake modelling tool such as PyWake^36^. Our workflow can be applied iteratively, to predict the power output of all farms in a given build-out scenario, and for a given set of weather conditions. Further detail regarding each of the components of the machine learning workflow is given in the next subsections. The Python code implementing this workflow is available via Code Ocean^55^.

#### Neural network farm wake model

We train a neural network to predict the wake deficit field induced by a wind farm, given the ‘background’ wind conditions. The wake deficit field *d* is defined as

1 $$d=\frac{| {{{\bf{u}}}}_{{{\rm{f}}}}| -| {{{\bf{u}}}}_{{{\rm{bg}}}}| }{| {{{\bf{u}}}}_{{{\rm{bg}}}}| },$$

where **u**_bg_ is the two-dimensional ‘background’ horizontal velocity field (with no farms present), and **u**_f_ is the velocity field with a wind farm. **u**_bg_ can be extracted from a WRF run with no wind farms, and **u**_f_ extracted from a WRF run with a wind farm. Power prediction requires the wind velocities at the WRF level closest to the relevant turbine hub height. We employ five ‘standard’ turbine types, and therefore compute deficit fields at the five WRF vertical levels closest to each hub height. The five turbines we select have rated powers of 2, 5, 8, 10.6, and 15 MW, similar to the range observed in the wind farm dataset. For each turbine, we obtain power curves from the NREL Turbine Archive^60^.

The neural network is trained to predict wake deficit fields which have been projected onto a ‘standard’ grid via rotation and cropping, such that the wind farm centroid is at coordinates *x* = 0, *y* = 0, and the wake is aligned with the positive *x* axis. The same rotation and cropping is applied to the neural network input data. The rotation is based on the background wind direction at the farm location at the final simulation timestamp, and hence does not utilise any information about the wake itself. This pre-processing step simplifies the wake prediction problem, using the a priori knowledge that wakes form in the downstream direction. The standard grid spans *y*-coordinates -64 to 64 km and *x*-coordinates of -40 to 152 km at a resolution of 4 km, and thus has dimensions 32 × 48. This modest spatial extent, along with the rotation pre-processing step, has the effect of removing much of the diversity in the input wind fields, and resulting wake deficit fields, which would otherwise arise due to the complexity of wind patterns over a large study area. This minimises the number of samples required to adequately train the neural network model, and increases the generalisation abilities of the trained model. Predicted deficit fields are mapped back onto the original WRF model grid by padding with zeros, and rotation (i.e. the inverse of the rotation and cropping pre-processing step).

The neural network itself utilises a U-Net architecture, consisting of convolutional layers, which are capable of taking advantage of the structured grid onto which the input wind fields have been transformed. The network has 32 input layers, each of shape 32 × 48, normalised on a per-layer basis to the range 0–1. 27 of these layers correspond to physical fields extracted from the ‘background’ WRF simulation and projected onto the ‘standard’ grid. These are the *u*, *v* and TKE fields at different vertical levels and simulation timestamps. TKE is assumed to act as a suitable proxy for the effects of atmospheric stability on wake formation; see section S-4 in the Supplementary Information for further discussion on this point. The remaining five input layers encode the number of turbines within each transformed grid cell, for each of the five turbine types (one layer per turbine type). While this representation of each farm’s turbines neglects any potential influence from the precise layout on sub-grid scales, the overall shape and density of the farm is well represented. This gridded approach is also consistent with that taken in the WRF model with the Fitch wind farm parameterisation, which we use to generate training data within this work. This input data is represented as a tensor of dimensions (32 × 48 × 32), i.e. using 32 channels. The U-Net architecture consists of four encoder levels, with 512 channels at each level, followed by four equivalent decoder levels. The neural network output is the velocity deficit field at the final timestamp *t* (as defined by equation (1)), at each of the five selected vertical levels. This is represented as a tensor of shape (32 × 48 × 5), which is again normalised on a per-layer basis to the range 0–1.

#### Neural network training

Each neural network training sample is generated in the form of a pair of model runs, with and without a wind farm, and each training model run spans a three-hour window in order to provide the ‘background’ wind fields and wake deficit field as described above. This data, along with the turbine layout, provides the input and target output for the neural network.

Training a neural network requires a large volume of training data, and we must therefore select a set of wind farms and weather conditions from which to generate training samples. We select all farms from build-out stages A and D, a total of 92 farms. Further detail regarding these farms is provided in Section S-2 of the Supplementary Information. For each, we take wind speed data from ERA-5 at 6-hourly intervals from the year 2000. From this, we perform stratified sampling, independently for each farm, to select up to 72 weather snapshots which capture the full range of wind conditions experienced by each farm. These samples form the basis for the neural network training. Since each farm is in a different location, and the neural network inputs are derived from the wind field local to the farm, each training sample therefore experiences a different wind field. For each sample, we run WRF for a three-hour window, using a domain configuration with three nested domains. The outer domain is the same as for the full North Sea runs described above, with the nested domains both centred on the wind farm, and spanning 810 and 450 km on each side, with resolutions of 6 and 2 km, respectively. This reduces the computational cost compared with running with either of the larger-scale configurations we use for generating the ‘background’ wind fields. Once this training data is generated, we further augment the dataset by reflecting each training sample in the *x* axis after projecting onto the ‘standard’ grid. This produces physically plausible wind fields and wakes, while still having the wake aligned with the *x* axis. This results in a total of 11,666 training samples.

Validation data, which is used for the purposes of early stopping within the training algorithm, is produced in a similar manner to the training dataset above. We use stratified sampling to select six validation samples for each farm. This data is again augmented as for the training dataset, yielding a total of 1094 samples.

To train the neural network, we use a mean squared error loss function. We use an Adam optimiser for network training with a learning rate of 5 × 10^−6^, a batch size of 5, and early stopping after 100 epochs without validation improvement. These parameters, along with the network architecture itself, were chosen following a preliminary hyperparameter sweep. The final parameter values are taken as those which produced the lowest value of the validation loss. The network is trained on an NVIDIA A100 GPU.

#### Superposition of farm wakes

The next step is the superposition of wakes from several farms. In the context of so-called engineering models for individual turbine wakes, a number of approaches for superposition have been used^62^; common methods include maximum deficit superposition e.g.^63^, linear superposition e.g.^64^, and root-sum-square superposition e.g.^65^. Through preliminary comparisons of these common turbine superposition methods, we found that linear superposition (i.e. simple summation of the deficit fields as defined by equation (1)) performs best in the context of inter-farm wakes. However, more sophisticated methods will be the topic of future work, to ensure that any complex and non-linear inter-farm interactions are adequately captured.

#### Farm power estimation

The variable of interest for the analysis of farm build-out, and for spatial planning, is the power output from a given farm in a given wind field (which may or may not be influenced by the wakes of other nearby farms). To estimate this, we take the wind speed at each turbine location and use the turbine power curve to estimate the power output, summing over the turbines present in each farm. This neglects intra-farm wake effects. A more sophisticated approach, taking account of intra-farm wakes, would be to use an off-the-shelf intra-farm model such as PyWake^36^. However, the focus of the current work is on quantifying and mitigating inter-farm wake effects. Preliminary experiments showed the inter-farm wake-induced losses were not sensitive to the choice of intra-farm model (see Supplementary Information Section S-9). The power curve approach is more computationally efficient, and thus is better suited to the pairwise analysis, sensitivity analysis and optimisation experiments we perform.

### Pairwise percentage losses

In the main text we introduced the Pairwise Percentage Loss (PPL), as well the the mutual PPL, as a useful measure of the strength of interaction between a given pair of farms. We denote the mean power output from a farm *F*_*i*_ in the presence of other farms {*F*_*k*_} as *P*(*F*_*i*_, {*F*_*k*_}). The mean power loss at farm *F*_*i*_ due to farm *F*_*j*_ is then given by

2 $${L}_{ij}=P({F}_{i},\{{F}_{k}\forall k\})-P({F}_{i},\{{F}_{k}\forall k\;\ne \; j\}).$$

The pairwise percentage loss (PPL) experienced by farm *F*_*i*_ due to farm *F*_*j*_ is then defined as

3 $${{{\rm{PPL}}}}_{ij}=100\times \frac{{L}_{ij}}{{{{\rm{AEP}}}}_{i}},$$

where AEP_*i*_ is the annual energy produced by farm *F*_*i*_ in the absence of any inter-farm wakes.

The mutual PPL is further defined as

4 $$\,{{\rm{Mutual\; PPL}}}({F}_{i},{F}_{j})=100\times \frac{{L}_{ij}+{L}_{ji}}{{{{\rm{AEP}}}}_{i}+{{{\rm{AEP}}}}_{j}}.$$

Figure 6 analysed the dependence of the PPL on farm separation distance, and angle with respect to the local prevailing wind. For this, the local prevailing wind is defined as the direction of the mean wind velocity.

## Supplementary information

Supplementary Information

## Data Availability

An example dataset is available on Code Ocean^55^. This study used reanalysis data from ERA-5 for model initial and boundary conditions^56^, which is publicly available via the Copernicus Climate Data Store (https://cds.climate.copernicus.eu). Further geographic input data was derived from the WRF Preprocessing System^57^. The full datasets generated and/or analysed during the current study are available from the corresponding author on reasonable request.

## Code availability

The WRF and PyWake software suites are publicly available from https://github.com/wrf-model/WRF and https://github.com/DTUWindEnergy/PyWake, respectively. Code implementing the modelling workflow, including the machine learning wake model, is available on Code Ocean^55^.

## References

[1] GWEC. Global Wind Energy Council Global Offshore Wind Report 2023 (2023).

[2] 4C Offshore. https://map.4coffshore.com/offshorewind/. Accessed: 2023-03-15.

[3] European Commission. An EU Strategy to harness the potential of offshore renewable energy for a climate neutral future (2020).

[4] NSEC & UK. Memorandum of Understanding on offshore renewable energy cooperation between the participants of the North Seas Energy Cooperation (NSEC), of the one side, and the United Kingdom of Great Britain and Northern Ireland, of the other side. https://www.gov.uk/government/publications/offshore-renewables-resources-in-the-north-seas-region-memorandum-of-understanding (2022).

[5] Platis, A. et al. First in situ evidence of wakes in the far field behind offshore wind farms. *Sci. Rep.* **8**, 1–10 (2018). 29391440 10.1038/s41598-018-20389-y PMC5794966

[6] Schneemann, J., Rott, A., Dörenkämper, M., Steinfeld, G. & Kühn, M. Cluster wakes impact on a far-distant offshore wind farm’s power. *Wind Energy Sci.* **5**, 29–49 (2020).

[7] Cañadillas, B. et al. Offshore wind farm wake recovery: Airborne measurements and its representation in engineering models. *Wind Energy* **23**, 1249–1265 (2020).

[8] Li, R., Zhang, J. & Zhao, X. Long-distance and high-impact wind farm wake effects revealed by SAR: a global-scale study. *arXiv preprint arXiv:2311.18124* (2023).

[9] Abkar, M. & Porté-Agel, F. Influence of atmospheric stability on wind-turbine wakes: A large-eddy simulation study. *Phys. fluids* **27**, 035104 (2015).

[10] Foreman, R. J., Cañadillas, B. & Robinson, N. The atmospheric stability dependence of far wakes on the power output of downstream wind farms. *Energies* **17**, 488 (2024).

[11] Nygaard, N. G. Wakes in very large wind farms and the effect of neighbouring wind farms. In *Journal of Physics: Conference Series*, vol. 524, 012162 (IOP Publishing, 2014).

[12] Mayol, M., Saulo, A. & Otero, A. Farm to farm wake interaction in WRF: impact on power production. In *Journal of Physics: Conference Series*, vol. 1934, 012017 (IOP Publishing, 2021).

[13] Fischereit, J. et al. Comparing and validating intra-farm and farm-to-farm wakes across different mesoscale and high-resolution wake models. *Wind Energy Sci.* **7**, 1069–1091 (2022).

[14] Energiewende, A. & Verkehrswende, A. Making the most of offshore wind: Re-evaluating the potential of offshore wind in the German North Sea. Agora Energiewende1-81 (2020).

[15] Fischereit, J., Brown, R., Larsén, X. G., Badger, J. & Hawkes, G. Review of Mesoscale Wind-Farm Parametrizations and Their Applications. *Bound.-Layer. Meteorol.* **182**, 175–224 (2022).

[16] Lundquist, J., DuVivier, K., Kaffine, D. & Tomaszewski, J. Costs and consequences of wind turbine wake effects arising from uncoordinated wind energy development. *Nat. Energy* **4**, 26–34 (2019).

[17] Finserås, E., Anchustegui, I. H., Cheynet, E., Gebhardt, C. G. & Reuder, J. Gone with the wind? wind farm-induced wakes and regulatory gaps. *Mar. Policy* **159**, 105897 (2024).

[18] Soares-Ramos, E. P., de Oliveira-Assis, L., Sarrias-Mena, R. & Fernández-Ramírez, L. M. Current status and future trends of offshore wind power in Europe. *Energy* **202**, 117787 (2020).

[19] Akhtar, N., Geyer, B., Rockel, B., Sommer, P. S. & Schrum, C. Accelerating deployment of offshore wind energy alter wind climate and reduce future power generation potentials. *Sci. Rep.* **11**, 1–12 (2021). 34083704 10.1038/s41598-021-91283-3 PMC8175401

[20] Warder, S. C. & Piggott, M. D. The future of offshore wind power production: Wake and climate impacts. *Appl. Energy* **380**, 124956 (2025).

[21] Pryor, S. C. & Barthelmie, R. J. Wind shadows impact planning of large offshore wind farms. *Appl. Energy* **359**, 122755 (2024).

[22] Skamarock, W. C. et al. A description of the advanced research WRF model version 4. *Natl. Cent. Atmos. Res.: Boulder, CO, USA* **145**, 145 (2019).

[23] Ali, K., Schultz, D. M., Revell, A., Stallard, T. & Ouro, P. Assessment of five wind-farm parameterizations in the Weather Research and Forecasting model: A case study of wind farms in the North Sea. Monthly Weather Review (2023).

[24] Fitch, A. C. et al. Local and mesoscale impacts of wind farms as parameterized in a mesoscale NWP model. *Monthly Weather Rev.* **140**, 3017–3038 (2012).

[25] Volker, P. J., Badger, J., Hahmann, A. N. & Ott, S. The Explicit Wake Parametrisation V1. 0: a wind farm parametrisation in the mesoscale model WRF. *Geoscientific Model Dev.* **8**, 3715–3731 (2015).

[26] Abkar, M. & Porté-Agel, F. A new wind-farm parameterization for large-scale atmospheric models. Journal of Renewable and Sustainable Energy 7 (2015).

[27] Pan, Y. & Archer, C. L. A hybrid wind-farm parametrization for mesoscale and climate models. *Bound.-Layer. Meteorol.* **168**, 469–495 (2018).

[28] Redfern, S., Olson, J. B., Lundquist, J. K. & Clack, C. T. Incorporation of the rotor-equivalent wind speed into the weather research and forecasting model’s wind farm parameterization. *Monthly Weather Rev.* **147**, 1029–1046 (2019).

[29] Jiménez, P. A., Navarro, J., Palomares, A. M. & Dudhia, J. Mesoscale modeling of offshore wind turbine wakes at the wind farm resolving scale: A composite-based analysis with the Weather Research and Forecasting model over Horns Rev. *Wind Energy* **18**, 559–566 (2015).

[30] Pryor, S. C., Shepherd, T. J., Volker, P. J., Hahmann, A. N. & Barthelmie, R. J. "Wind Theft” from onshore wind turbine arrays: sensitivity to wind farm parameterization and resolution. *J. Appl. Meteorol. Climatol.* **59**, 153–174 (2020).

[31] Pryor, S. C., Barthelmie, R. J. & Shepherd, T. J. Wind power production from very large offshore wind farms. *Joule* **5**, 2663–2686 (2021).

[32] Cuevas-Figueroa, G., Stansby, P. K. & Stallard, T. Accuracy of WRF for prediction of operational wind farm data and assessment of influence of upwind farms on power production. *Energy* 124362 (2022).

[33] Cañadillas, B. et al. Offshore wind farm cluster wakes as observed by long-range-scanning wind lidar measurements and mesoscale modeling. *Wind Energy Sci.* **7**, 1241–1262 (2022).

[34] Pryor, S. C. & Barthelmie, R. J. Power production, inter-and intra-array wake losses from the us east coast offshore wind energy lease areas. *Energies* **17**, 1063 (2024).

[35] Dörenkämper, M., ollmer, L. & Lakdawala, Z. Simulating the impact of offshore wind expansion on yield and efficiency in the north sea by 2050https://www.bsh.de/DE/THEMEN/Offshore/Meeresfachplanung/_Anlagen/Downloads/IWES_Bericht.pdf (2025).

[36] Pedersen, M. M., van der Laan, P., Friis-Møller, M., Rinker, J. & Réthoré, P.-E. DTUWindEnergy/PyWake: PyWake10.5281/zenodo.2562662 (2019).

[37] NREL. FLORIS Wake Modeling and Wind Farm Controls Software. https://github.com/NREL/floris (2022).

[38] Park, J. & Park, J. Physics-induced graph neural network: An application to wind-farm power estimation. *Energy* **187**, 115883 (2019).

[39] Ti, Z., Deng, X. W. & Zhang, M. Artificial Neural Networks based wake model for power prediction of wind farm. *Renew. Energy* **172**, 618–631 (2021).

[40] Li, R., Zhang, J. & Zhao, X. Dynamic wind farm wake modeling based on a Bilateral Convolutional Neural Network and high-fidelity LES data. *Energy* **258**, 124845 (2022).

[41] Wang, L. et al. Effectiveness of data-driven wind turbine wake models developed by machine/deep learning with spatial-segmentation technique. *Sustain. Energy Technol. Assess.* **53**, 102499 (2022).

[42] Li, S., Zhang, M. & Piggott, M. D. End-to-end wind turbine wake modelling with deep graph representation learning. *Appl. Energy* **339**, 120928 (2023).

[43] Stieren, A. & Stevens, R. J. Evaluating wind farm wakes in large eddy simulations and engineering models. In *Journal of physics: Conference series*, vol. 1934, 012018 (IOP Publishing, 2021).

[44] Dörenkämper, M. & Steinfeld, G. Wind Farm Cluster Wakes. In *Handbook of Wind Energy Aerodynamics*, 1039-1076 (Springer, 2022).

[45] Nygaard, N. G., Steen, S. T., Poulsen, L. & Pedersen, J. G. Modelling cluster wakes and wind farm blockage. In *Journal of Physics: Conference Series*, vol. 1618, 062072 (IOP Publishing, 2020).

[46] Pedersen, J., Svensson, E., Poulsen, L. & Nygaard, N. Turbulence optimized park model with Gaussian wake profile. In *Journal of Physics: Conference Series*, vol. 2265, 022063 (IOP Publishing, 2022).

[47] Machiridza, L. & Bhattacharya, S. Levelized cost of energy (UK offshore wind power) drivers, challenges, opportunities and practice 2010–20. Wind Energy Engineering501-526 (2023).

[48] Martinez, A. & Iglesias, G. Mapping of the levelised cost of energy for floating offshore wind in the European Atlantic. *Renew. Sustain. Energy Rev.* **154**, 111889 (2022).

[49] Gad, A. F. Pygad: An intuitive genetic algorithm python library. *Multimed. tools Appl.* **83**, 58029–58042 (2024).

[50] Mosetti, G., Poloni, C. & Diviacco, B. Optimization of wind turbine positioning in large windfarms by means of a genetic algorithm. *J. Wind Eng. Ind. Aerodyn.* **51**, 105–116 (1994).

[51] Azlan, F., Kurnia, J., Tan, B. & Ismadi, M.-Z. Review on optimisation methods of wind farm array under three classical wind condition problems. *Renew. Sustain. Energy Rev.* **135**, 110047 (2021).

[52] GWEC. Global Wind Energy Council Global Wind Report 2025 (2025).

[53] Department for Energy Security & Net Zero. National Policy Statement for Renewable Energy Infrastructure (EN-3) https://assets.publishing.service.gov.uk/media/6809f0588c1316be7978e7cb/draft-nps-en-3.pdf/ (2025).

[54] Warder, S. C. & Piggott, M. D. Mapping global offshore wind wake losses, layout optimisation potential, and climate change effects. *Energy* 136573 (2025).

[55] Warder, Simon C and Clare, Mariana C A and Bhaskaran, B and Piggott, Matthew D. Code ocean repository: Assessment and optimisation of regional scale wind farm deployment using machine learning 10.24433/CO.6804660.v1 (2026). 10.1038/s44172-026-00673-w PMC13333893 42056455

[56] Hersbach, H. et al. The ERA5 global reanalysis. *Q. J. R. Meteorological Soc.* **146**, 1999–2049 (2020).

[57] NCAR/MMM. WRF Preprocessing System (WPS) Geographical Static Data Repository. https://www2.mmm.ucar.edu/wrf/users/download/get_sources_wps_geog.html (2021). Accessed: 2022-07-08.

[58] Friedl, M. A. et al. Global land cover mapping from MODIS: algorithms and early results. *Remote Sens. Environ.* **83**, 287–302 (2002).

[59] Friedl, M. A. et al. MODIS Collection 5 global land cover: Algorithm refinements and characterization of new datasets. *Remote Sens. Environ.* **114**, 168–182 (2010).

[60] NREL Turbine Archive. https://nrel.github.io/turbine-models/index.html (2023). Accessed: 2023-04-04.

[61] Fitch, A. C. Notes on using the mesoscale wind farm parameterization of Fitch et al.(2012) in WRF. *Wind Energy* **19**, 1757–1758 (2016).

[62] Vogel, C. R. & Willden, R. H. Investigation of wind turbine wake superposition models using Reynolds-averaged Navier-Stokes simulations. *Wind Energy* **23**, 593–607 (2020).

[63] Larsen, G. C., Madsen, H. A., Thomsen, K. & Larsen, T. J. Wake meandering: a pragmatic approach. *Wind Energy.: Int. J. Prog. Appl. Wind Power Convers. Technol.* **11**, 377–395 (2008).

[64] Lissaman, P. Energy effectiveness of arbitrary arrays of wind turbines. *J. Energy* **3**, 323–328 (1979).

[65] Katic, I., Højstrup, J. & Jensen, N. O. A simple model for cluster efficiency. In *European wind energy association conference and exhibition*, vol. 1, 407-410 (A. Raguzzi Rome, Italy, 1986).

