# Supplementary material for: Assessment and optimisation of regional scale wind farm deployment using machine learning
Source: Commun Eng. 2026 Apr 29;5:121. doi: 10.1038/s44172-026-00673-w (PMC13333893; doi:10.1038/s44172-026-00673-w)
Supplement: Supplementary file 1 — Supplementary Information [file 44172_2026_673_MOESM1_ESM.pdf]

Supplementary Information

S-1 Underlying wind resource in the North Sea

Figure S-1 shows the mean underlying wind power density in the North Sea, based on ERA-5 wind speed data at 100 m, spanning the years 2000–2019. The wind power density is given by

$$P = \frac{1}{2}\rho_{\mathrm{air}}|\mathbf{u}|^3, \tag{S-1}$$

where ρ_{air} is the density of air (taken as 1.225 $\mathrm{kg\,m^{-3}}$), and $\mathbf{u}$ is the wind velocity at a given height. Figure S-1 is qualitatively consistent with resource maps such as the New European Wind Atlas[1;2].

S-2 Wind farm data

As described in the main text, we group wind farms according to a simple criterion. Farms separated by more than 2 km or 12 rotor diameters are considered to be separate entities, with any closer pairs of farms grouped together. This ensures that closely coordinated wind farm clusters are treated as a single entity. The motivation for this choice is that we are interested in exploring the negative impacts between uncoordinated wind farm developments. We therefore make two exceptions to this criterion. The first is to retain separation between the 'East Anglia ONE' and 'East Anglia ONE North' farms. These two farms do not form a tessellating cluster, they comprise differing turbine technologies, and differ substantially in their capacity density per unit area. The second exception is to retain separation between 'Hollandse Kust Noord' and 'Egmond aan Zee', for similar reasons; these farms have vastly differing technologies, designs and capacity densities, and do not form a tessellating cluster.

Figure S-2 summarises the distribution of turbine types used for neural network training within this study. The number of farms with each turbine type ranges from nine (for 2 MW turbines) to 26 (for 5 and 15 MW turbines). Despite therefore having relatively small numbers of training samples corresponding to each individual turbine type, we find that the neural network performs well. We found through preliminary experiments that a single network trained using multiple turbine types (with one input channel per type) performed better than dividing the training data by turbine type and training a separate network for each. This suggests that the neural network is able to extract key information

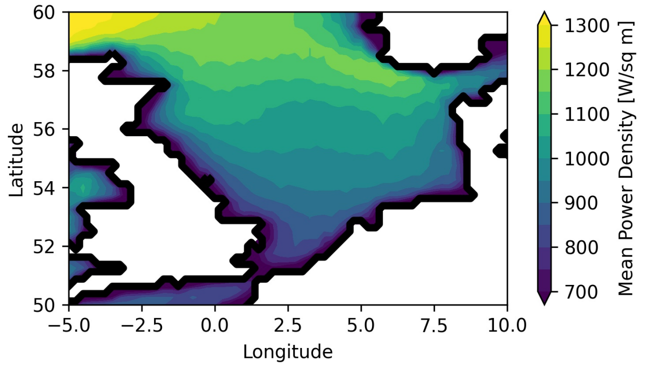

Figure S-1: Mean power density in the North Sea, based on wind velocity at 100 m during the 20-year period used within this study. All data presented within this figure is derived from open-access ERA-5 data[3].

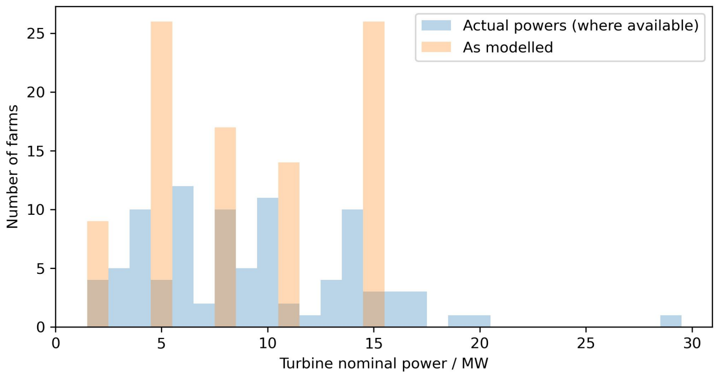

Figure S-2: Distribution of turbine types for North Sea farms used for neural network training within this study.

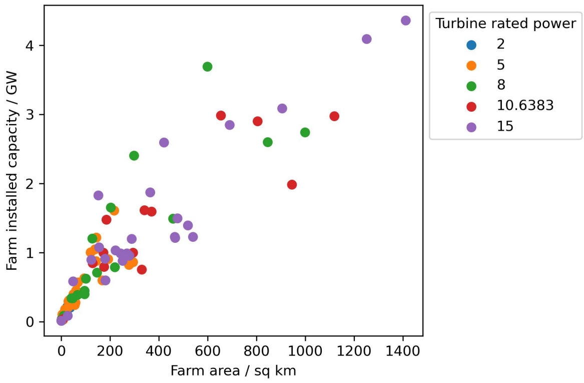

Figure S-3: Spatial area and installed capacity of North Sea farms used for neural network training within this study.

from the turbine input channels, which is then propagated through the rest of the network in a turbine-agnostic way. We therefore expect that transfer learning could be used to extend this framework to further turbines, with minimal new training data. However, this is beyond the scope of this study and left for future work.

Figure S-3 shows the spatial areas, installed capacities and turbine types of the farms used for training. The median installed capacity density is 5.9 MW km^{-2}, which is consistent with typical values for North Sea wind farms[4]. The data spans a large range of wind farm sizes and installed capacities. We can also see visually from Figure 1 in the main text that the wind farms span a variety sizes, shapes and aspect ratios.

S-3 Neural network model resolution

Figure S-15 shows the distribution of the median turbine separation distance for all farms at build-out stage D. The smallest is 0.36 km, and the largest is 3.25 km. This supports the choice to use a resolution of 4 km for the neural network wake model. At a finer resolution such as 2 km (the same as the underlying WRF model resolution), farms with greater turbine separations would be represented by sparse turbine density maps, creating a more challenging modelling problem for the neural network. At 4 km, this issue is avoided, while maintaining a reasonably high fidelity in the simulated wakes and avoiding very large numbers of turbines per grid cell (we find an average of 5.0 turbines per 4 km grid cell, excluding empty cells).

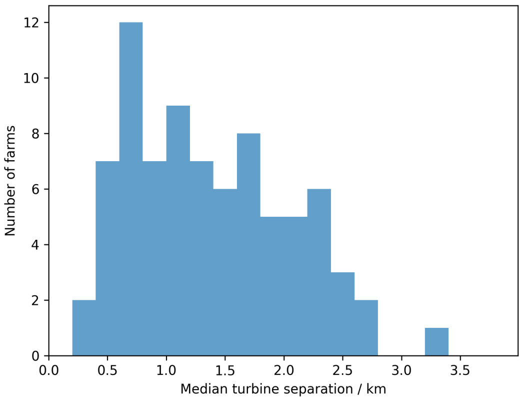

Figure S-4: Distribution of the median turbine separation distance for each farm at build-out stage D.

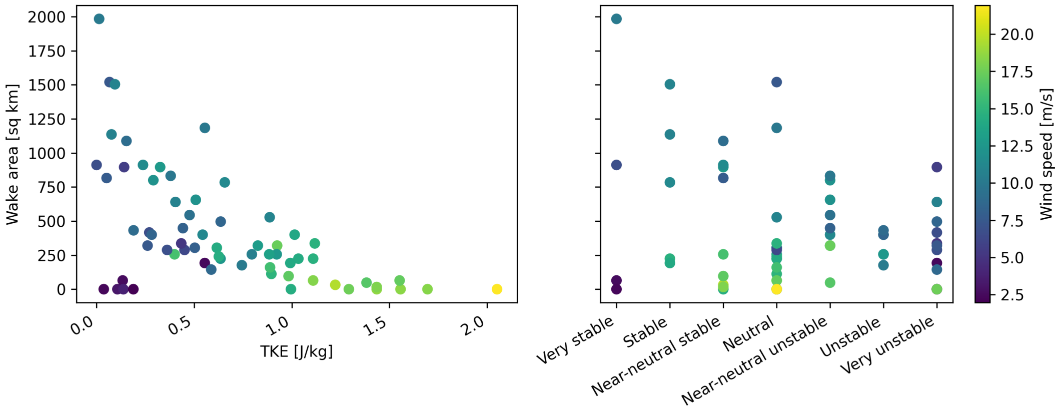

Figure S-5: Dependence of wake area (defined as the area of the region where wake deficits exceed 5%) on TKE (left) and atmospheric stratification regime (right). Stratification regimes are based on the Monin-Obukhov length[7]. Data is taken from the neural network training data, for the East Anglia ONE wind farm. There is a clear dependence of wake area on TKE, with higher TKE strongly suppressing wake extent. Very low wind speeds explain the outliers at low TKE. There is no clear direct relationship between wake area and stratification.

S-4 Influence of atmospheric stratification

Atmospheric stratification is known to have a large impact on wake recovery[5;6]. A key mechanism for this is mixing induced by increased turbulent kinetic energy (TKE) under unstable conditions. As summarised in figure S-5 using WRF-generated training data from a selected wind farm, TKE constitutes a powerful explanatory variable for wake extent (defined as the area of the region where wake deficits exceed 5% in magnitude), while direct correlation with stratification regime is relatively weak. For the purposes of this study, TKE was therefore assumed to be a suitable proxy for the effects of atmospheric stratification on wake dynamics. The inclusion of further proxies for atmospheric stability will be considered in future work.

S-5 Model training

Figure S-6 summarises the training of the neural network model, showing the training and validation losses as a function of the number of training epochs. We find that the validation loss starts to level out after around 100–150 epochs. Although the training loss continues to decrease after this point, the validation loss remains stable, suggesting that the model performance is not suffering due to overfitting. Overfitting is further mitigated by the use of early stopping; the optimisation is stopped after 100 epochs without any improvement in the validation loss. The final model parameters are taken as those which

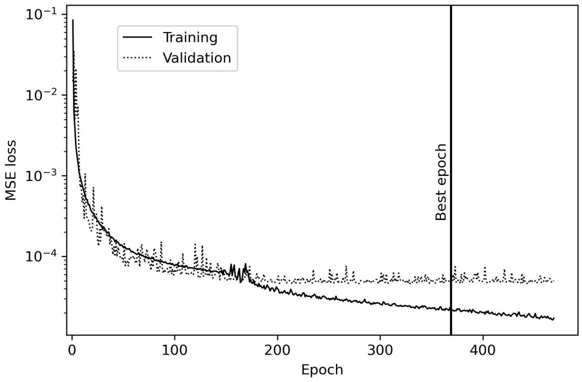

Figure S-6: Training and validation loss (mean squared error) during model training.

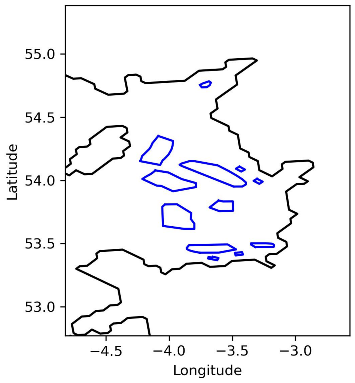

Figure S-7: Wind farms in the Irish Sea, used in this study as the basis for an independent test dataset. Geographic data within this figure is derived from the MODIS land use dataset [8;9], which is an open access dataset and distributed as static geographic data via the WRF Preprocessing System (WPS) [10].

minimised the validation loss, which was achieved after 369 epochs. For the final trained model, the training loss is 2.2×10^{-5}, and the validation loss is 4.6×10^{-5}.

S-6 Testing on unseen farms

In this section we test the generalisation ability of our wake modelling framework. To achieve this, we present results from applying our model to unseen samples, which consist of new wind farms, in new locations, and in new wind fields. The wind farms are situated in the eastern Irish Sea. Following the same approach as described in the main text for the North Sea, we take all operational and planned wind farms, equivalent to build-out stage D from the North Sea study. This produces a set of 12 farms as shown in figure S-7, which span a variety of spatial areas, installed capacities and turbine types, as summarised in figure S-8.

The wind conditions we use to generate the test dataset are drawn in the same way as described for the validation dataset in the main text, using stratified sampling to select six samples for each farm, spanning a range of wind speeds and directions. We choose to sample from the year 1999 to generate this test dataset (whereas the training and validation datasets were generated based on the year 2000). Though not strictly necessary in order to demonstrate model generalisation (since farms in different locations always experience different local wind conditions), this ensures that our model is indeed generalising to new wind fields.

The mean squared error for this test dataset is 4.1×10^{-5}. This is in fact smaller than for the validation dataset (4.6×10^{-5}). We also present example wake deficit fields in figure S-9, where we observe good agreement between the wakes as modelled in WRF, and via our machine learning workflow.

These results demonstrate that our farm wake model is capable of making accurate predictions for new farms in new regions, with unseen wind conditions. We attribute this good generalisation performance

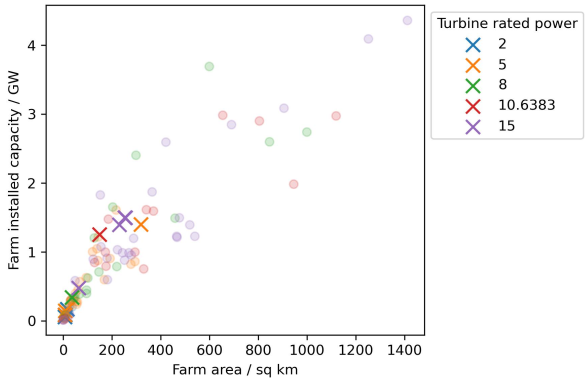

Figure S-8: Spatial area and installed capacity of Irish Sea farms used for model validation, shown as crosses. Shown in the background are the training farms from the North Sea.

to a number of design choices.

Firstly, we note that the use of 92 wind farms for training may be considered small compared with the number of degrees of freedom in the turbine map inputs, which have a shape of $32 \times 48 \times 5$. However, the rotation preprocessing step in our workflow, which ensures that all winds input into the neural network are propagating in the positive x-direction, means that the precise turbine density map corresponding to each of the 72 training samples generated per wind farm will sum to the same value, but will not be identical. This is particularly pronounced for wind farms with a high aspect ratio.

The use of convolutional layers also aids in model generalisation. With convolutional layers, the number of trainable parameters depends on the kernel size, rather than the input size. This means that, although the use of spatially varying fields results in model inputs with around 50,000 degrees of freedom, we are still able to train effectively with only around 10,000 training samples.

S-7 Sensitivity analysis and optimisation experiments

The fixed set of perturbations applied to each wind farm within the sensitivity analysis and optimisation experiments is shown in figure S-10. They are based on perturbation distances of 3.5, 7, 10 and 12 km, and four perturbation directions for each distance. In total there are 17 possible perturbations (including the unperturbed location). These possible perturbations are sampled randomly for each farm within the sensitivity analysis experiments described in the main text. The range of perturbations is an arbitrary choice. However, we consider the perturbations to be sufficiently small that the resulting configuration remains plausible, while our results show that they are large enough to facilitate a substantial improvement in OWF fleet performance.

We emphasise that performing these experiments via standard numerical modelling would require an unfeasible number of numerical model runs (1,000 samples $\times$ 1,392 weather conditions). However, using the neural network workflow, this becomes computationally feasible.

The *a priori* set of perturbations is also used as the basis for the genetic optimisation algorithm. We use the PyGAD Python package to implement the genetic optimisation algorithms[11]. The fitness score is the percentage increase in total fleet power relative to the baseline (unperturbed) build-out design. Each generation of the algorithm consists of 16 members, and the mutation rate is 10%. The initial population is selected randomly. We run the optimisation until 10 generations pass without improvement in the generation's best fitness score.

For the full North Sea case study in the main text, we split the domain into three subdomains, as shown in figure S-11, and perform the optimisation on each subdomain separately. This was due to memory constraints on the machine used for the optimisation, and the fact that the required memory scales linearly with both the domain area and number of farms (or with the square of the domain area assuming uniform density of farms). We assume that farms only influence other farms within the same subdomain. This is a reasonable assumption since the shortest distance between farms assigned to separate subdomains is 117 km, which exceeds typical wake lengths, especially since the farms in question are relatively small. The second closest farms are 170 km apart. Interactions over distances greater than around 150 km are in any case neglected by the workflow, due to the spatial extent of the 'standard' grid

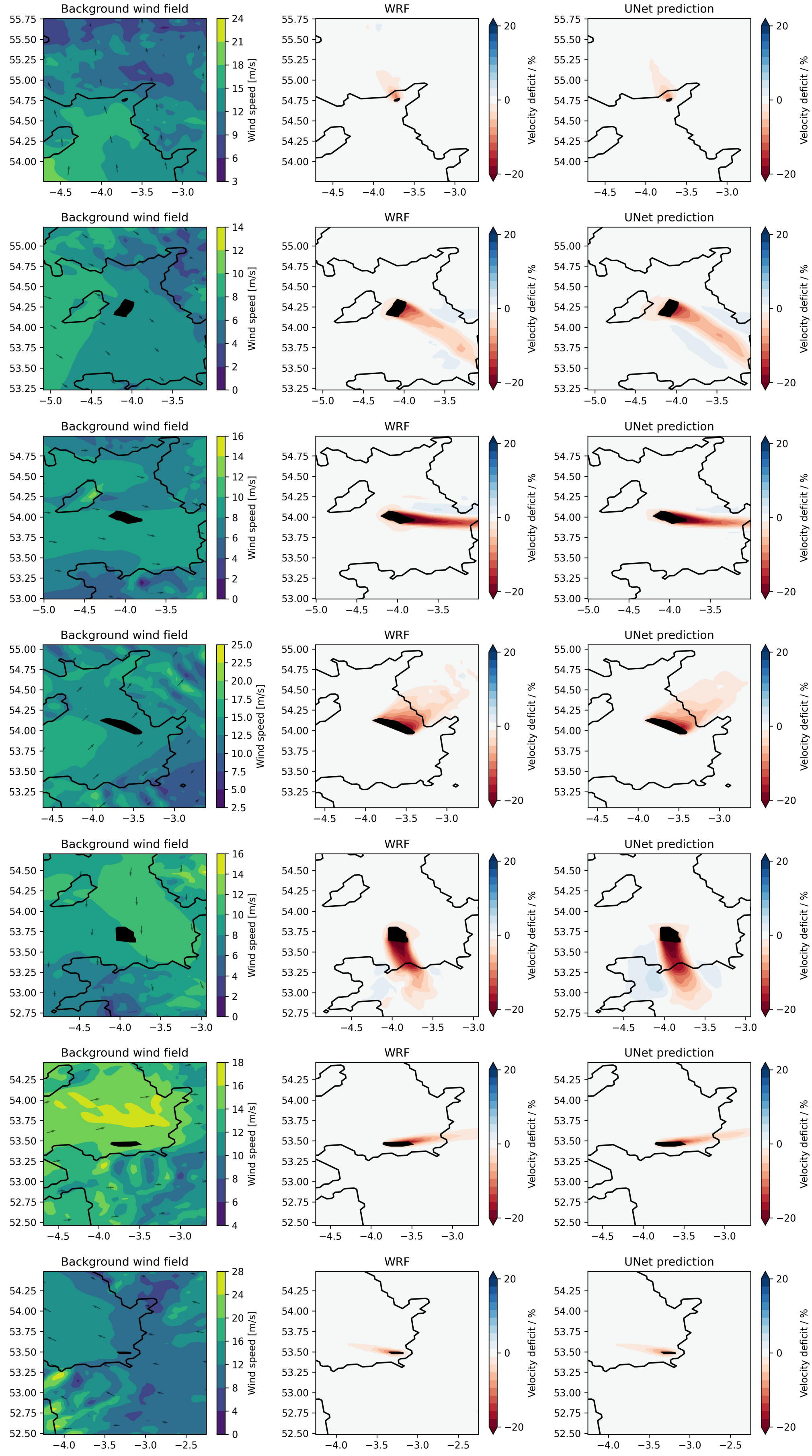

Figure S-9: A selection of wind and wake deficit fields, for unseen test cases in the Irish Sea. Geographic data within this figure is derived from the MODIS land use dataset [8;9], which is an open access dataset and distributed as static geographic data via the WRF Preprocessing System (WPS) [10].

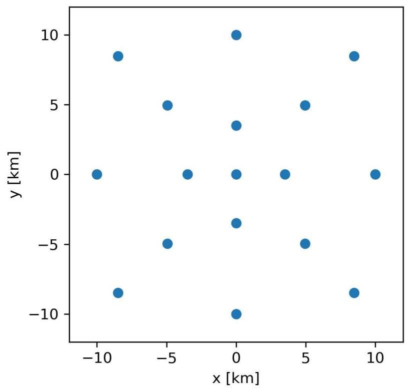

Figure S-10: Perturbations applied to farm locations within sensitivity analysis/optimisation case study.

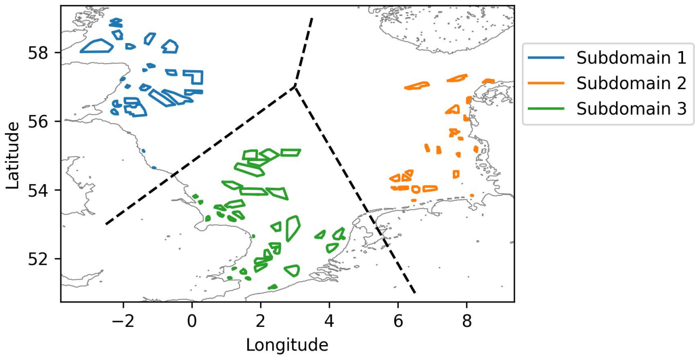

Figure S-11: Subdomains for optimisation of full North Sea case study. Geographic data within this figure is derived from the MODIS land use dataset [8;9], which is an open access dataset and distributed as static geographic data via the WRF Preprocessing System (WPS) [10].

used for the neural network. The subdivision of the domain for the purposes of optimisation therefore has negligible impact on the results of our sensitivity and optimisation study.

S-8 Southern North Sea case study

S-8.1 Sensitivity analysis & optimisation

In the main text, we applied the sensitivity analysis and optimisation workflow to the entire North Sea. Here we show results from a smaller-scale case study in the Southern North Sea, consisting of the Thames Estuary and surrounding regions. For this case study we use only a single year of weather conditions, from 2001, again spaced 5.25 days apart (a total of 70 snapshots). The small spatial domain and reduced set of weather snapshots facilitate validation of selected build-out configurations using full NWP simulations.

The Thames Estuary study region contains 18 wind farms. Of these, 11 are already operational and thus held constant. The remaining seven farms are permitted to move by up to 12 km, as in the full North Sea study in the main text, and described above.

The results are shown in figure S-12. The wake-free power varies by up to 0.62% relative to the baseline design, and the wake-induced losses vary between -3.96 and -2.19%. The resulting relative waked power varies between -1.22 and 1.05%. The optimal design produces 1.50% greater waked power than the baseline design. This is driven by both greater wake-free power (i.e. the farms are located in slightly windier conditions on average), and improved wake-induced losses.

Figure S-13 shows the mutual PPLs in the Thames Estuary region for the planned build-out, and

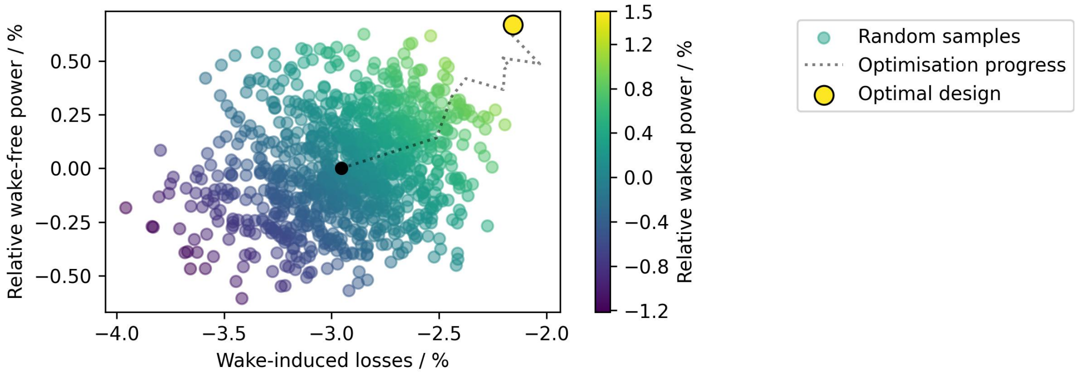

Figure S-12: Scatter plot showing the relationship between the wake-free power, the wake-induced losses, and the waked power, for the Thames Estuary region. Powers are measured as percentage changes relative to the baseline design. The waked power is due to the combination of the wake-free power and the wake-induced losses. The random samples have relative waked power values of between -1.22 and 1.05%. The optimal design has a relative waked power of 1.50%, achieving both a greater wake-free power, and better wake-induced losses, than any of the random samples.

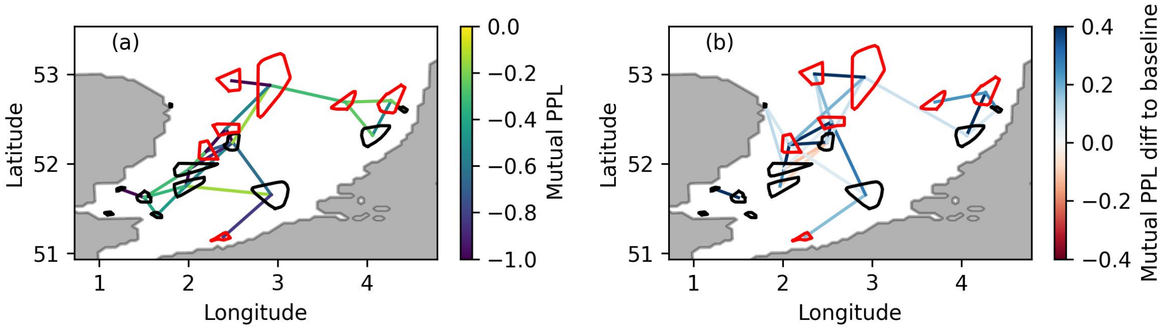

Figure S-13: (a) Mutual pairwise percentage losses (PPLs) between pairs of farms in the Thames Estuary case study region, for planned build-out locations. (b) Best-case build-out locations, and changes in mutual PPL relative to the planned build-out. Fixed farms are shown in black, free farms in red. Positive mutual PPL differences indicate improved wake-induced losses, and only mutual PPL differences with an absolute value greater than 0.04% are shown. Several mutual PPLs see large improvements, while there are no mutual PPLs which worsen substantially. Geographic data within this figure is derived from the MODIS land use dataset[8;9], which is an open access dataset and distributed as static geographic data via the WRF Preprocessing System (WPS)[10].

the changes in the mutual PPL as a result of the optimal build-out. Note that, since only the year 2001 was used for this analysis, the planned mutual PPLs differ slightly from those in figure 5 of the main text, which were based on the full period spanning the years 2000–2019. There are only four mutual PPL values whose change between the baseline and optimised layouts is less than -0.02%, while there are 15 mutual PPLs which improve by at least 0.1%. Overall, only three farms produce less power in the optimal layout than in the baseline layout, with the greatest power reduction equal to 0.21%. All three of the farms whose output is reduced in the optimal design are existing operational farms, whose locations were held constant within the optimisation. 15 farms produce more power in the optimal layout, with 10 of these increasing output by 1% or more. The reduction in overall losses is therefore achieved without a substantial negative impact to any individual farm.

Using figure S-13 to compare the planned and optimal layouts, it is clear that the improved performance of the optimal configuration is largely achieved by simply increasing inter-farm separation distances. However, we find that the angle between any given pair of farms relative to the prevailing wind also has an influence on mutual PPL. The relative strength of the distance and angle effects depends on the pair of farms in question.

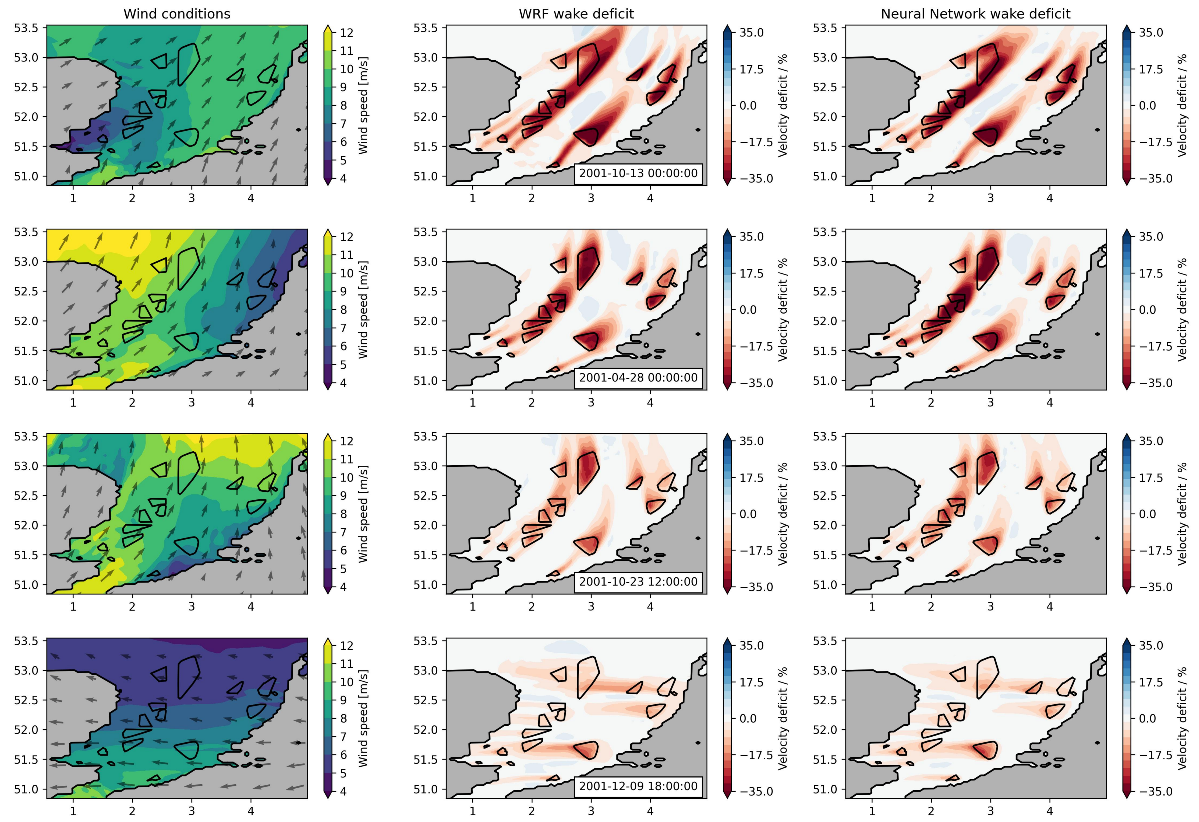

Figure S-14: Comparison of wake deficit fields in the Thames Estuary region, as predicted using WRF and the neural network workflow, for randomly selected weather snapshots at times as indicated. Geographic data within this figure is derived from the MODIS land use dataset [8;9], which is an open access dataset and distributed as static geographic data via the WRF Preprocessing System (WPS) [10].

S-8.2 Validation using full NWP simulations

S-8.2.1 Wake deficit field comparisons

We first present a comparison of wake deficits in the region, for build-out stage D. The aim is to verify that the wake prediction and superposition components of the workflow are producing wake fields consistent with those predicted using WRF. Figure S-14 shows wake deficit fields as predicted by the neural network and from WRF simulations, for a selection of wind conditions. The wake shapes and intensities are well predicted by the neural network, including speedup effects around and between farms. Figure S-15 compares the mean deficit fields over the year 2001, as predicted via the neural network and WRF. We again find that the neural network captures the spatial variability in the deficit intensity.

S-8.2.2 Power deficit comparisons

Having verified that the neural network-derived wake deficit fields are representative of those simulated using WRF, in this section we compare power estimates from the neural network + power curve workflow (which we refer to as NN+PC) with those derived from WRF, to assess whether the overall workflow produces useful power estimates.

The first experiment is based on the planned build-out scenario. The objective is to further validate the wake prediction and superposition stage of the neural network workflow. We therefore compare power estimates from the NN+PC workflow, with those based on a WRF+PC workflow. To obtain WRF+PC power estimates, WRF must be run once not only for each weather snapshot, but also for each wind farm in the region. This is because, to compute the power output from a particular farm using the power curve method, the wind field input into the power curves should not include the farm's own wake. WRF simulations are therefore required to exclude each farm, one at a time; since there are 70 weather snapshots and 18 farms, this requires a total of $70 \times 18 = 1,260$ runs. Once the wake-affected wind fields are simulated using WRF, we use the power curves in a manner consistent with the NN+PC workflow.

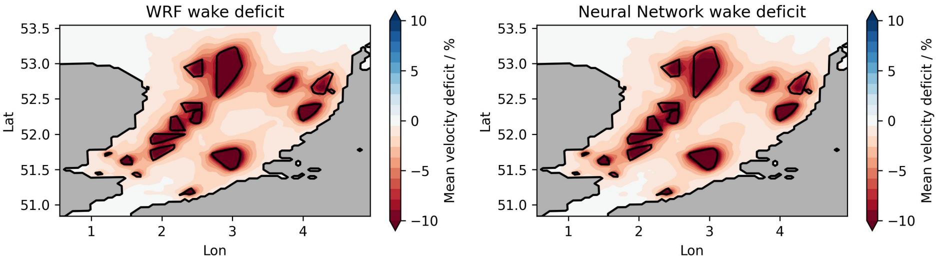

Figure S-15: Comparison of mean deficit fields in the Thames Estuary region, as predicted using WRF and the neural network workflow. Geographic data within this figure is derived from the MODIS land use dataset[8;9], which is an open access dataset and distributed as static geographic data via the WRF Preprocessing System (WPS)[10].

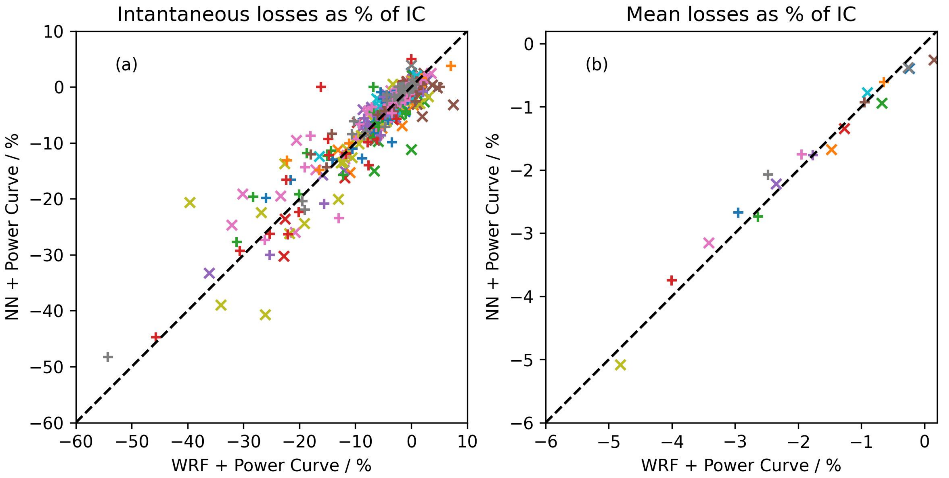

Figure S-16: (a): Comparison of instantaneous (i.e. per weather snapshot) wake-induced losses, for each farm in the Thames Estuary study, based on planned build-out locations. Each colour and marker shape combination corresponds to a different farm. The RMSE is 2.05%, and the R^2 coefficient is 0.85. (b): Comparison of mean (over all weather snapshots in the year 2001) wake-induced losses, for each farm. The RMSE is 0.22% and the R^2 coefficient is 0.98.

The results from this validation experiment are shown in figure S-16. There is good agreement between the NN+PC and WRF+PC power estimates. The RMSE of the instantaneous (per-weather snapshot) losses is 2.05%, and for the mean losses the RMSE is 0.22%. This good agreement implies that the NN-predicted wakes are of sufficient accuracy to be used to produce useful estimates of wake-induced power losses.

The second validation experiment is based on comparing power estimates from the full NN+PC workflow, with those obtained directly from WRF. In this case, the WRF model need only be run once per weather snapshot, since we do not need to pass WRF wind fields into a second power model. This reduced computational cost facilitates a comparison for multiple build-out designs; we compare power estimates for 23 different farm configurations. The selected designs correspond to the baseline and optimised designs, plus the 0th–100th percentile wake-induced losses, in steps of 5%. The results are shown in figure S-17. Since the power estimation methods differ, figure S-17 compares the percentage changes in total mean power relative to the baseline design rather than absolute power values. We observe good agreement, with an R^2 coefficient of 0.95. Note that although WRF also uses power curves to estimate farm power, some intra-farm effects are still accounted for, in cases where farms span multiple grid cells. Hence any discrepancies in figure S-17 have contributions from both the neural network wake predictions, and the different treatment of intra-farm effects. The good agreement demonstrates that build-out designs optimised using the NN+PC workflow also perform well in full WRF simulations.

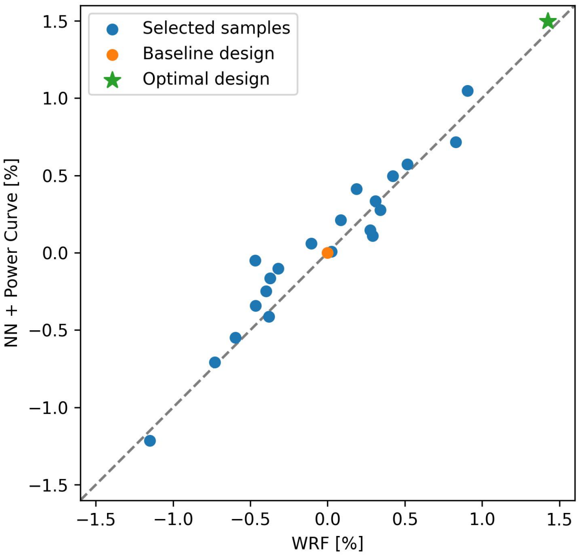

Figure S-17: Estimated percentage changes in total power output (relative to the planned build-out) within the Thames Estuary study, for a selection of wind farm layouts from the random samples. Here, the WRF values are based on the use of WRF for the entire validation workflow, including power estimates. The RMSE is 0.15 % and the R^2 coefficient is 0.95. This result indicates that comparing (or optimising) build-out designs using the neural network workflow is consistent with using full NWP simulations.

S-9 Comparison of intra-farm models

As noted in the main text, we choose to use a simple power curve method to estimate the power output of each wind farm, when subject to a particular wind field (which may or may not include the wake effect from another nearby farm). This approach incurs negligible computational cost, and is thus well suited to the applications we consider, where low computational cost is essential.

However, this approach neglects intra-farm wake effects, which in reality would further reduce power outputs. These losses could also be considered to be the negative influence of a wind farm on its own power production. In general this influence arises from both the wakes from individual turbines within a farm reducing the wind speeds experienced by their neighbours, and also farm-scale effects such as blockage[12]. Since the objectives of this study are to quantify and minimise losses due to inter-farm wakes, we can justify neglecting intra-farm effects, as long as the estimated inter-farm losses do not depend on our treatment of intra-farm effects, i.e. that the cumulative impact of inter- and intra-farm effects is reasonably approximated as being linear.

The experiments in this section are intended to verify that our estimates of inter-farm wake-induced losses do not depend on our treatment of intra-farm wakes. To investigate this, we couple our workflow to an industry-standard intra-farm modelling tool. The model we choose for comparison is PyWake[13], and specifically the IEA37SimpleBastankhahGaussianDeficit wake model[14], with the self-similarity blockage model[15] (updated 2020 version) and mirror ground model, as recommended by Forsting et al.[16] for capturing turbine and farm blockage effects. The coupling procedure involves estimating the inter-farm wake-affected wind fields as per our primary workflow, which are then used as input to a PyWake-based model of the relevant wind farm.

We present a comparison of power output results from the two methods (PyWake and the simple power curve approach) for the Thames Estuary case study at build-out stage D, using the planned build-out locations. Figure S-18 compares the instantaneous and mean power output from each method, as a percentage of the installed capacity of each farm (commonly referred to as the capacity factor). The power curve method produces larger power estimates than PyWake. This is because PyWake accounts for intra-farm wake effects, reducing power, and the power curve method does not. This difference is most pronounced for capacity factors of around 0.5, since this is where a turbine's power output is most sensitive to the wind speed, and therefore to intra-farm wakes. However, figure S-19 compares the instantaneous and mean inter-farm wake-induced losses for the two power estimation methods. Despite the overestimation of power output from the power curve method, the two methods produce good agreement

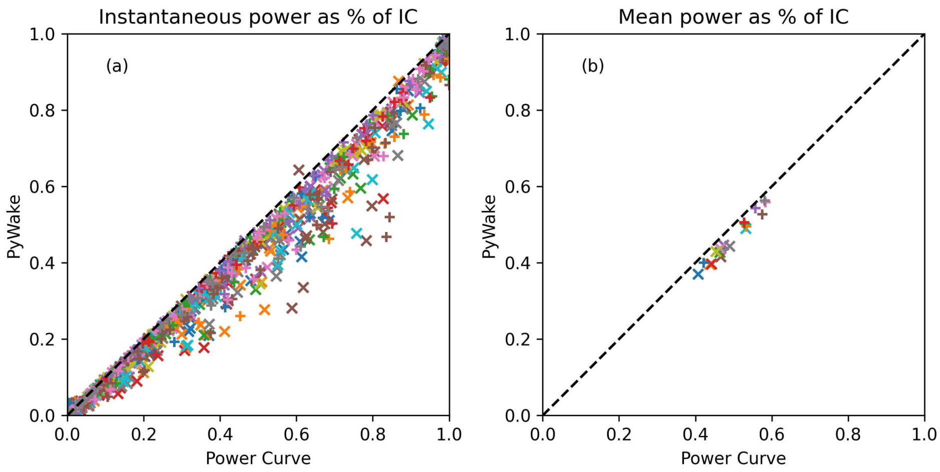

Figure S-18: Comparison of instantaneous power as computed using PyWake and the simple power curve method, for the Thames Estuary case study. Each colour and marker style combination indicates a different wind farm. Since PyWake accounts for intra-farm wake effects and the power curve method does not, the power computed by PyWake is in almost all cases lower than that returned by the power curve.

in the losses due to inter-farm wakes. Since the focus of this work is on these losses, this result indicates that the use of a simple power curve approach is sufficient.

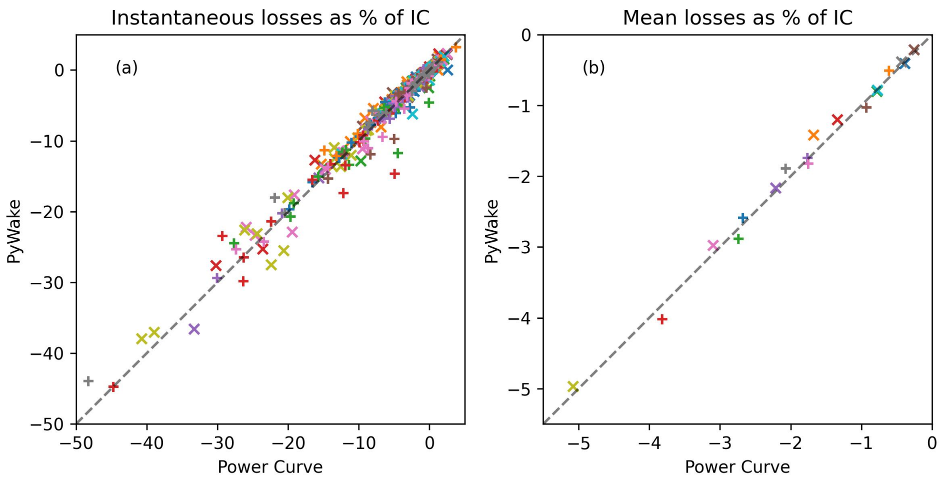

Figure S-19: Comparison of (a) instantaneous and (b) mean inter-farm wake-induced losses, as computed using PyWake and the simple power curve method, for the Thames Estuary case study. Each colour and marker style combination indicates a different wind farm. For the instantaneous losses, the RMSE is 0.74% and the R^2 coefficient is 0.98. For the mean losses, the RMSE is 0.12% and the R^2 coefficient is 0.99. Although the power curve method overestimates power output compared with PyWake (as shown in figure S-18), there is good agreement in the losses due to inter-farm wakes.

References

[1] Andrea N Hahmann, Tija Sīle, Björn Witha, Neil N Davis, Martin Dörenkämper, Yasemin Ezber, Elena García-Bustamante, J Fidel González-Rouco, Jorge Navarro, Bjarke T Olsen, et al. The making of the New European Wind Atlas–part 1: model sensitivity. *Geoscientific model development*, 13 (10):5053–5078, 2020. doi: https://doi.org/10.5194/gmd-13-5053-2020.

[2] Martin Dörenkämper, Bjarke T Olsen, Björn Witha, Andrea N Hahmann, Neil N Davis, Jordi Barcons, Yasemin Ezber, Elena García-Bustamante, J Fidel González-Rouco, Jorge Navarro, et al. The making of the new european wind atlas–part 2: Production and evaluation. *Geoscientific model development*, 13(10):5079–5102, 2020. doi: https://doi.org/10.5194/gmd-13-5079-2020.

[3] Hans Hersbach, Bill Bell, Paul Berrisford, Shoji Hirahara, András Horányi, Joaquín Muñoz-Sabater, Julien Nicolas, Carole Peubey, Raluca Radu, Dinand Schepers, et al. The ERA5 global reanalysis. *Quarterly Journal of the Royal Meteorological Society*, 146(730):1999–2049, 2020. doi: https://doi.org/10.1002/qj.3803.

[4] Rehfeld Borrmann, K Rehfeldt, AK Wallasch, and Silke Lüers. Capacity densities of European offshore wind farms. *Deutsche WindGuard GmbH*, 2018.

[5] Mahdi Abkar and Fernando Porté-Agel. Influence of atmospheric stability on wind-turbine wakes: A large-eddy simulation study. *Physics of fluids*, 27(3):035104, 2015. doi: https://doi.org/10.1063/1.4913695.

[6] Richard J Foreman, Beatriz Cañadillas, and Nick Robinson. The atmospheric stability dependence of far wakes on the power output of downstream wind farms. *Energies*, 17(2):488, 2024. doi: https://doi.org/10.3390/en17020488.

[7] Ameya Sathe, J Mann, Julia Gottschall, and MS Courtney. Estimating the systematic errors in turbulence sensed by wind lidars. *Risø National Laboratory, Roskilde, Denmark. Risø*, page 24, 2010.

[8] Mark A Friedl, Douglas K McIver, John CF Hodges, Xiaoyang Y Zhang, Douglas Muchoney, Alan H Strahler, Curtis E Woodcock, Sucharita Gopal, Annemarie Schneider, Amanda Cooper, et al. Global land cover mapping from MODIS: algorithms and early results. *Remote sensing of Environment*, 83 (1-2):287–302, 2002. doi: https://doi.org/10.1016/S0034-4257(02)00078-0.

[9] Mark A Friedl, Damien Sulla-Menashe, Bin Tan, Annemarie Schneider, Navin Ramankutty, Adam Sibley, and Xiaoman Huang. MODIS Collection 5 global land cover: Algorithm refinements and characterization of new datasets. *Remote sensing of Environment*, 114(1):168–182, 2010. doi: https://doi.org/10.1016/j.rse.2009.08.016.

[10] NCAR/MMM. WRF Preprocessing System (WPS) Geographical Static Data Repository. https://www2.mmm.ucar.edu/wrf/users/download/get_sources_wps_geog.html, 2021. Accessed: 2022-07-08.

[11] Ahmed Fawzy Gad. PyGAD: An Intuitive Genetic Algorithm Python Library, 2021.

[12] James Bleeg, Mark Purcell, Renzo Ruisi, and Elizabeth Traiger. Wind farm blockage and the consequences of neglecting its impact on energy production. *Energies*, 11(6):1609, 2018. doi: https://doi.org/10.3390/en11061609.

[13] Mads M. Pedersen, Paul van der Laan, Mikkel Friis-Møller, Jennifer Rinker, and Pierre-Elouan Réthoré. DTUWindEnergy/PyWake: PyWake, February 2019. URL https://doi.org/10.5281/zenodo.2562662.

[14] Majid Bastankhah and Fernando Porté-Agel. A new analytical model for wind-turbine wakes. *Renewable energy*, 70:116–123, 2014. doi: https://doi.org/10.1016/j.renene.2014.01.002.

[15] Niels Troldborg and Alexander Raul Meyer Forsting. A simple model of the wind turbine induction zone derived from numerical simulations. *Wind Energy*, 20(12):2011–2020, 2017. doi: https://doi.org/10.1002/we.2137.

[16] Alexander R Meyer Forsting, Gonzalo P Navarro Diaz, Antonio Segalini, Søren J Andersen, and Stefan Ivanell. On the accuracy of predicting wind-farm blockage. *Renewable Energy*, 2023. doi: https://doi.org/10.1016/j.renene.2023.05.129.
